# CD38 promotes pristane-induced chronic inflammation and increases susceptibility to experimental lupus by an apoptosis-driven and TRPM2-dependent mechanism

**DOI:** 10.1038/s41598-018-21337-6

**Published:** 2018-02-20

**Authors:** Sonia García-Rodríguez, Antonio Rosal-Vela, Davide Botta, Luz M. Cumba Garcia, Esther Zumaquero, Verónica Prados-Maniviesa, Daniela Cerezo-Wallis, Nicola Lo Buono, José-Ángel Robles-Guirado, Salvador Guerrero, Elena González-Paredes, Eduardo Andrés-León, Ángel Corbí, Matthias Mack, Friedrich Koch-Nolte, Ramón Merino, Mercedes Zubiaur, Frances E. Lund, Jaime Sancho

**Affiliations:** 1 Department of Cellular Biology and Immunology, IPBLN-CSIC, Granada, Spain; 2 Department of Microbiology, UAB, Birmingham, Alabama USA; 3 Flow Cytometry Unit, IPBLN-CSIC, Granada, Spain; 4 Bioinformatics Unit, IPBLN-CSIC, Granada, Spain; 5 Department of Molecular Microbiology and Infection Biology, CIB-CSIC, Madrid, Spain; 6 Department of Internal Medicine II, Nephrology, Regensburg University Medical Center, Regensburg, Germany; 7 Institute of Immunology, University Medical Center Eppendorf-Hamburg, Hamburg, Germany; 8 Department of Molecular and Cellular Signalling, IBBTEC-CSIC-UC, Santander, Spain; 9 Present Address: Immunology Graduate Program, Mayo Clinic, Rochester, MN USA; 10 Present Address: Melanoma Group, CNIO, Madrid, Spain; 11 Present Address: Laboratory of Immune-mediated Diseases, San Raffaele Diabetes Research Institute (DRI), Milano, Italy

## Abstract

In this study, we investigated the role of CD38 in a pristane-induced murine model of lupus. CD38-deficient (*Cd38*^−/−^) but not ART2-deficient (*Art2*^−/−^) mice developed less severe lupus compared to wild type (WT) mice, and their protective phenotype consisted of (i) decreased IFN-I-stimulated gene expression, (ii) decreased numbers of peritoneal CCR2^hi^Ly6C^hi^ inflammatory monocytes, TNF-α-producing Ly6G^+^ neutrophils and Ly6C^lo^ monocytes/macrophages, (iii) decreased production of anti-single-stranded DNA and anti-nRNP autoantibodies, and (iv) ameliorated glomerulonephritis. *Cd38*^−/−^ pristane-elicited peritoneal exudate cells had defective CCL2 and TNF-α secretion following TLR7 stimulation. However, *Tnf-α* and *Cxcl12* gene expression in *Cd38*^−/−^ bone marrow (BM) cells was intact, suggesting a CD38-independent TLR7/TNF-α/CXCL12 axis in the BM. Chemotactic responses of *Cd38*^−/−^ Ly6C^hi^ monocytes and Ly6G^+^ neutrophils were not impaired. However, *Cd38*^−/−^ Ly6C^hi^ monocytes and Ly6C^lo^ monocytes/macrophages had defective apoptosis-mediated cell death. Importantly, mice lacking the cation channel TRPM2 (*Trpm2*^−/−^) exhibited very similar protection, with decreased numbers of PECs, and apoptotic Ly6C^hi^ monocytes and Ly6C^lo^ monocytes/macrophages compared to WT mice. These findings reveal a new role for CD38 in promoting aberrant inflammation and lupus-like autoimmunity via an apoptosis-driven mechanism. Furthermore, given the implications of CD38 in the activation of TRPM2, our data suggest that CD38 modulation of pristane-induced apoptosis is TRPM2-dependent.

## Introduction

Systemic lupus erythematosus (SLE) is a chronic multi-organ inflammatory disease in which autoantibodies against nucleic acid–protein complexes, such as chromatin and ribonucleoproteins (RNPs), cause disease by forming immune complexes that deposit in target tissues. A type I IFN–dependent lupus syndrome closely resembling human SLE develops in BALB/c, C57BL/6 (B6), and other strains of mice with chronic inflammation following an intraperitoneal injection of pristane (2,6,10,14-tetramethylpentadecane)^1,2,3^. Autoantibody production and glomerulonephritis in pristane-induced lupus require Toll-like receptor 7 (TLR-7)–mediated type I IFN production driven by the transcription factors IFN regulatory factor 5 (IRF-5) and IRF-7^3^.

CD38 is a type II transmembrane protein, which can function as a receptor or as an ectoenzyme^4–6^. In fact, CD38 is considered a multifunctional enzyme capable of recognizing nicotinamide adenine dinucleotide (NAD) and nicotinamide adenine dinucleotide phosphate (NADP) as substrates, and generating four products, namely nicotinamide, adenosine diphosphate ribose (ADPR), which is an activator of the redox-sensitive cation channel Transient Receptor Potential Melastatin 2 (TRPM2), and cyclic ADPR (cADPR) and nicotinic acid adenine dinucleotide phosphate (NAADP), which are potent Ca^2+^-mobilizing intracellular messengers. CD38 is expressed in hematopoietic and non-hematopoietic tissues, and has been shown to regulate neutrophil migration and DC trafficking^7–11^, and that this regulation occurred via the modulation of extracellular calcium by TRPM2^12^. CD38 ligation with anti-CD38 antibodies stimulates cell proliferation, adhesion, apoptosis and cytokine secretion in different cell types^6^. In human T and B cells, upon receptor stimulation CD38 leads to PKB/AKT and ERK activation^13,14^. In regulatory T cells (Tregs), expression of CD38 is regulated by phosphoinositide 3-kinase (PI3K) p110δ^15^. Furthermore, CD19^+^CD24^hi^ B cells that express high levels of CD38, and which exhibit regulatory capacity in healthy individuals, produced less IL-10 and lacked suppressive capacity in SLE patients^16^. Moreover, CD38 plays an important role in modulating antigen-mediated T-cell responses at the immunological synapse, where CD38 is actively recruited by a Lck-mediated mechanism^17^. CD38 is a pathogenic and prognostic marker in human leukemias (MM, B-CLL, AML)^18^.

Increased CD38 surface expression in SLE T cells is more prevalent in clinically active SLE patients, while high levels of anti-CD38 autoantibodies are more frequent in quiescent SLE patients with low levels of anti-dsDNA autoantibodies^19^. Likewise, increased mRNA expression of CD38 in SLE T cells correlates with increased severity of the disease, and is considered part of a NAD(P)-binding domain gene expression signature associated with SLE^20^. Increased CD38 expression in SLE T cells could be the consequence of the action of pro-inflammatory cytokines such as TNF-α and IFN-γ, and is indicative of SLE patients with a more active disease, and with an overt abnormal Th2 and Th1 cytokine profile^19^. On the other hand, it has been previously reported that CD38-deficient (*Cd38*^−/−^) mice develop an attenuated form of Collagen-induced arthritis (CIA) compared to WT mice^21^ that is accompanied by a limited joint induction of IL-1β and IL-6 expression, by the lack of induction of IFN-γ expression in the joints, and by a reduction in the percentages of invariant natural killer T (iNKT) cells in the spleen. Immunized *Cd38*^−/−^ mice produce high levels of circulating IgG1 and low levels of IgG2a and anti-collagen II antibodies, which correlate with reduced frequencies of Th1 cells in the draining lymph nodes. Therefore, CD38 participates in the pathogenesis of CIA by controlling the number of iNKT cells and promoting Th1 inflammatory responses^21^.

In the present study, we examined the role of CD38 during the early inflammatory phase of pristane-induced lupus, a murine model of systemic lupus erythematosus (SLE). We show that pristane-treated *Cd38*^−/−^ mice, but not *Art2*^−/−^ mice, had impaired recruitment of peritoneal exudate cells (PECs) compared to WT mice, particularly Ly6C^hi^ monocytes and Ly6C^lo^ monocytes/macrophages and Ly6G^+^ neutrophils, and that this decreased cellularity in the peritoneal cavity (PC) was not due to a defective chemotaxis of these cells but instead due to decreased apoptosis-mediated cell death of PECs, characterized by defective caspase-3 activation and decreased levels of phosphorylated Akt. We identified Ly6C^hi^ monocytes and Ly6C^lo^ monocytes/macrophages as the apoptosis-resistant cell populations. Given the implication of CD38 in the activation of the cation channel TRPM2 via the production of cADPR/ADPR, we analysed the phenotype of pristane-treated *Trpm2*^−/−^ mice, and observed similar protection from apoptosis-mediated death of PECs in these mice. Furthermore, the production of anti-single-stranded DNA and anti-nuclear RNP (nRNP) autoantibodies, the development of glomerulonephritis, and the expression of IFN-I-stimulated genes (ISGs) were also greatly attenuated in pristane-treated *Cd38*^−/−^ mice. Hence, our data suggest that CD38 deficiency protects mice from pristane-induced lupus in a TRPM2-dependent manner by reducing the number of intraperitoneal apoptotic cells, which are the primary source of autoantigens in this murine model of SLE.

## Results

### CD38 deficiency alters the chronic inflammatory response to pristane by an ART2-independent mechanism

**Figure 1:**
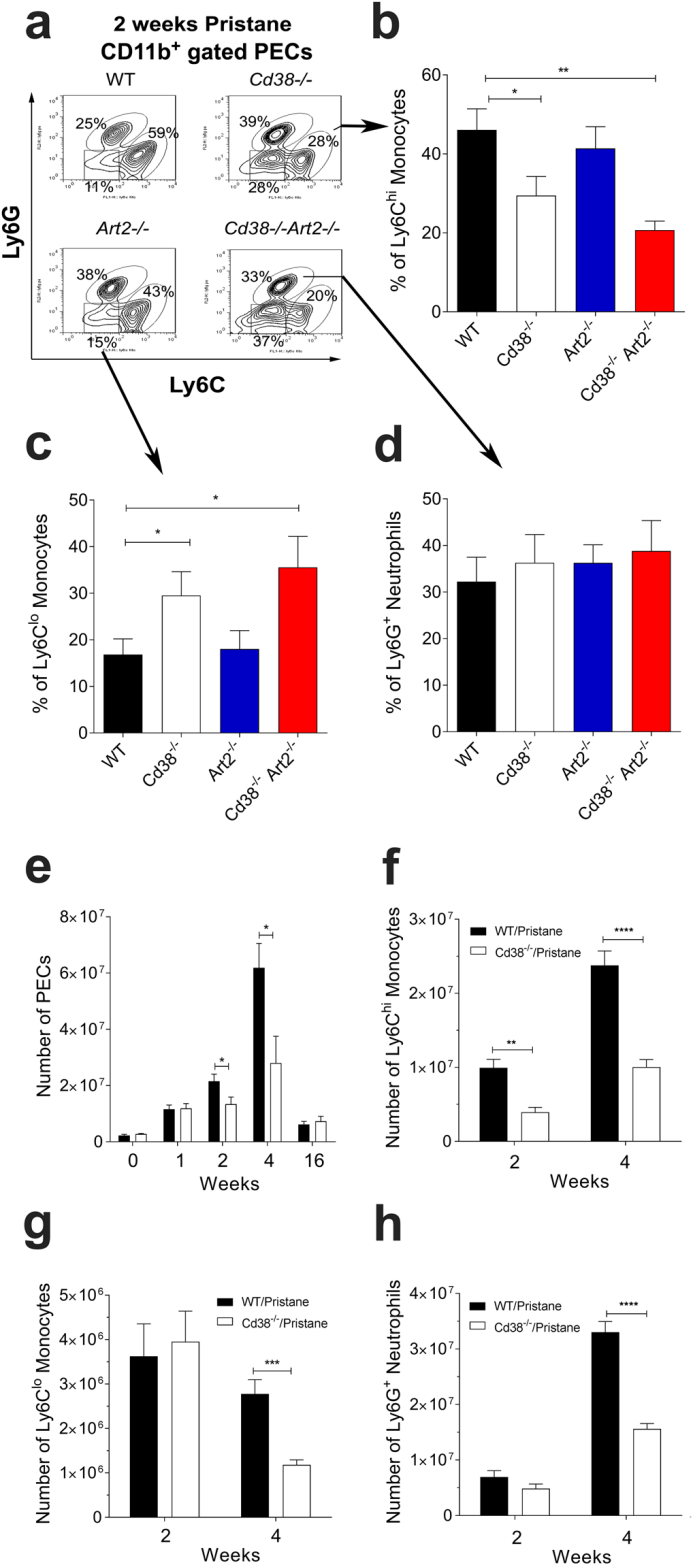
Altered pristane-induced chronic inflammation in *Cd38*^−/−^ mice. (**a**) Flow cytometric analysis of Ly6C and Ly6G expression on CD11b^+^-gated PECs at 2-weeks post pristane treatment. Contour plots show the frequencies of Ly6C^hi^ monocytes, Ly6G^+^ neutrophils, and Ly6C^lo^ monocytes/macrophages as a percentage of total CD11b^+^ PECs from WT, *Cd38*^−/−^, *Art2*^−/−^, and *Cd38*^−/−^*Art2*^−/−^ mice. Contour plots shown are representative of 3 independent experiments. (**b–d**) Frequencies of Ly6C^hi^ monocytes (**b**) Ly6C^lo^ monocytes/macrophages **(c)** and Ly6G^+^ neutrophils **(d)** as a percentage of total PECs from WT, *Cd38*^−/−^, *Art2*^−/−^, and *Cd38*^−/−^*Art2*^−/−^ mice. Data are shown as the mean ± SE (n = 7–9 mice/group). **(e)** Absolute numbers of total PECs at 0-, 1-, 2-, 4-, and 16-weeks post pristane treatment. Data are shown as mean ± SE (n = 5–14 mice/group). **(f–h)** Numbers of Ly6C^hi^ monocytes **(f)**, Ly6C^lo^ monocytes/macrophages **(g)** and Ly6G^+^ neutrophils **(h)** at 2- and 4-weeks post pristane treatment. Data are shown as the mean ± SE (n = 7–9 mice/group). All P values were determined by 2-way ANOVA with the Dunnett’s multiple comparisons test. *P < 0.05 **P < 0.01 ***P < 0.001 ****P < 0.0001.

It has previously been reported that a subset of inflammatory monocytes expressing high levels of CCR2 and Ly6C is recruited to the peritoneum in response to CCL2 (MCP-1) and is a major source of IFN-I in pristane-treated mice^22^. The effect of CD38 deficiency on the recruitment of inflammatory Ly6C^hi^ monocytes (CD11b^+^Ly6C^hi^Ly6G^−^), Ly6C^lo^ monocytes (CD11b^+^Ly6C^lo^Ly6G^−^), and neutrophils (CD11b^+^Ly6C^lo^Ly6G^+^) was examined 2 weeks following an intraperitoneal (i.p.) injection of pristane. Consistent with previous findings^22,23^, most pristane-elicited monocytes in WT mice expressed high levels of Ly6C (Fig. 1a). However, PECs from pristane-treated *Cd38*^−/−^ mice contained significantly lower frequencies and numbers of Ly6C^hi^ monocytes compared to pristane-treated WT controls (Fig. 1b). Moreover, PECs from *Cd38*^−/−^ mice contained a significantly higher frequency of Ly6C^lo^ monocytes compared to WT mice (Fig. 1c). The relative frequency of neutrophils, on the other hand, was comparable to that in WT mice (Fig. 1d).

NAD released during inflammation participates in the *in vivo* regulation of T cell homeostasis through an ART2-dependent and P2X7-mediated mechanism^24^, that is commonly referred to as NAD-induced cell death (NICD)^25^). NICD is controlled in part by CD38, which limits substrate availability for ART2 by hydrolysing extracellular NAD^26^. In the absence of CD38, ART2 preferentially activates apoptotic deletion of peripheral iNKT cells, especially the CD4^+^ subset, resulting in the reduction of NKT cells under steady-state conditions^21^. Since reduced numbers and/or functions of NKT cells have been shown to exacerbate pristane-induced lupus nephritis^27^, we assessed whether the absence of ART2, or the absence of both CD38 and ART2, had any influence on the persistent, pristane-induced inflammatory cell influx into the peritoneal cavity. ART2-deficient (*Art2*^−/−^) mice showed an intact influx of Ly6C^hi^ monocytes at 2-weeks post-pristane treatment that was similar to WT mice, whereas *Cd38*^−/−^*Art2*^−/−^ double knockout mice exhibited a decreased frequency of recruited Ly6C^hi^ monocytes that was similar to that observed in *Cd38*^−/−^ mice (Fig. 1b). Therefore, the defective pristane-induced influx of Ly6C^hi^ monocytes into the peritoneal cavity seems to be CD38-dependent but ART2-independent.

The pristane-induced influx of cells into the PC of *Cd38*^−/−^ and WT mice followed similar kinetics. Indeed, both groups of mice had increased numbers of PECs following pristane treatment, which peaked at 4 weeks and returned to basal levels by 16 weeks (Fig. 1e). No significant differences were observed in the number of PECs between the two groups of mice at 1-week post treatment, suggesting that *Cd38*^−/−^ mice exhibit an intact early response to pristane. However, at 2- and 4-weeks post treatment, the numbers of PECs were significantly lower in *Cd38*^−/−^ mice compared to WT mice, reaching similar numbers only at 16-weeks post treatment, when the inflammatory response is less severe and the symptoms of lupus begin to manifest^28^.

The differences in total numbers of PECs between pristane-treated WT and *Cd38*^−/−^ mice had a clear effect on the absolute numbers of the Ly6C^hi^ monocyte, Ly6C^lo^ monocyte/macrophage and Ly6G^+^ neutrophil cell subsets analysed. We observed a significantly lower number of Ly6C^hi^ monocytes in *Cd38*^−/−^ mice compared to WT mice at both 2-weeks and 4-weeks post pristane treatment (Fig. 1f), while decreased numbers of Ly6C^lo^ monocytes and Ly6G^+^ neutrophils in *Cd38*^−/−^ mice appeared only at 4-weeks post pristane (Fig. 1g and h). Hence, our data convincingly show that pristane induces a chronic inflammatory response in *Cd38*^−/−^ mice, albeit more attenuated than in WT mice.

### Pristane-elicited PECs from *Cd38*^−/−^ mice exhibit defective caspase-3 activation

**Figure 2:**
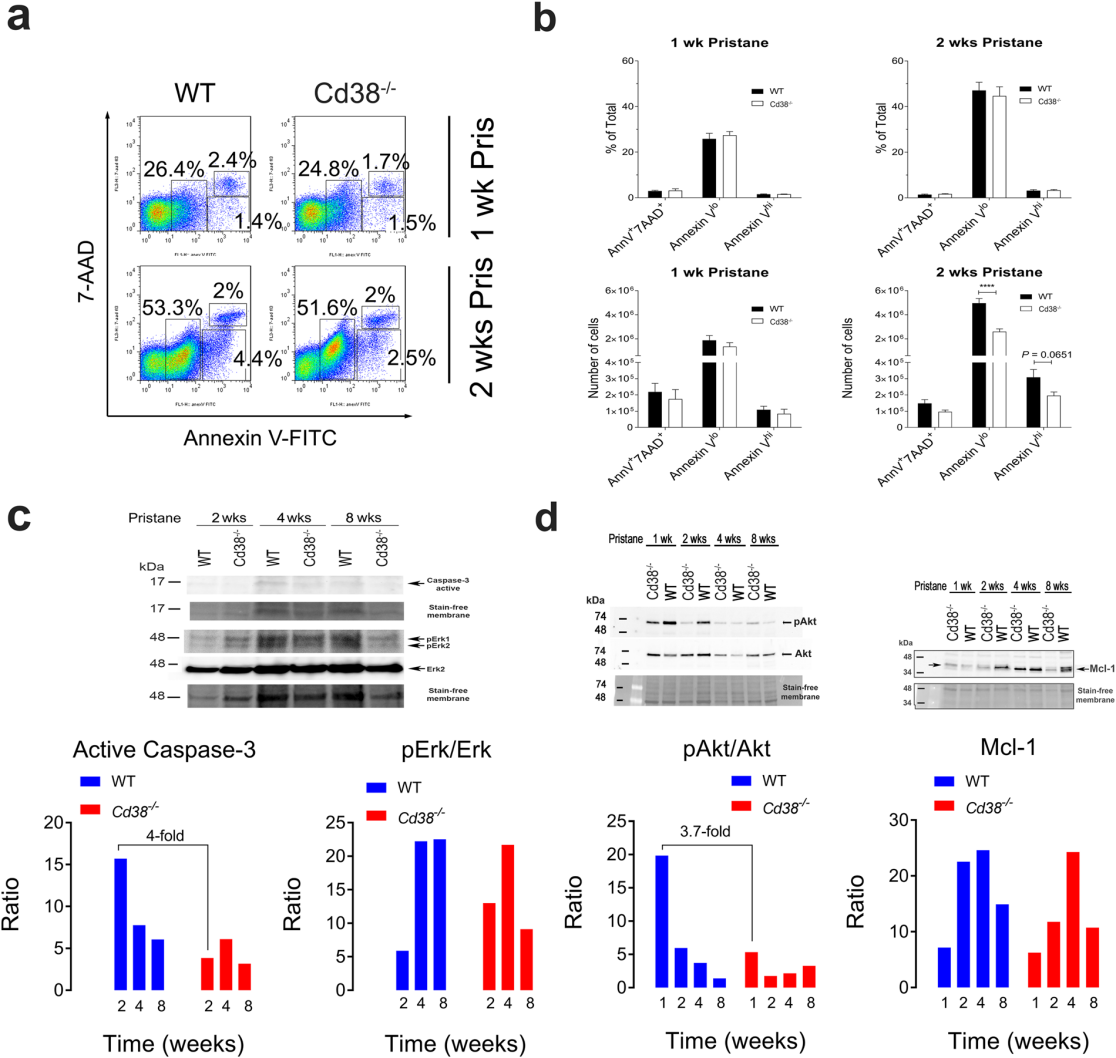
Defective caspase-3 activation in pristane-elicited PECs from *Cd38*^−/−^ mice. (**a**) Flow cytometric analysis of Annexin V (AnnV) and 7-AAD staining on PECs from 1-week pristane treated (upper panels) and 2-weeks pristane-treated (lower panels) WT and *Cd38*^−/−^ mice. Frequencies of early apoptotic (AnnV^lo^7-AAD^−^), late apoptotic (AnnV^hi^7-AAD^−^) and necrotic cells (AnnV^hi^7-AAD^+^) are shown. **(b**) Frequencies (top panels) and absolute numbers (bottom panels) of necrotic, early apoptotic, and late apoptotic PECs from 1- and 2-weeks pristane-treated WT and *Cd38*^−/−^ mice. Closed (WT) and open (*Cd38*^−/−^) bars represent the mean and the error bars indicate SE (n = 5-6 mice/group. P values were determined by 2-way ANOVA with the Bonferroni’s multiple comparisons test. ****P < 0.0001. **(c**) Lysates from 2-, 4- and 8-weeks pristane-elicited PECs were subjected to Western blotting using antibodies specific for active caspase-3, phospho-Erk1/2 (Thr^185^/Tyr^187^), total Erk1/2, and the corresponding stain-free areas of the PVDF membrane before blotting. Each lane represents a pool of 3 lysates from 3 mice/group. Densitometric analyses of the amount of active caspase-3 or phosphorylated Erk1/2 were done using the Image Lab software (Bio-Rad Laboratories). In the lower left and right panels the normalized amount of active caspase-3 and the pErk/Erk ratios are shown, respectively. **(d**) Lysates from 1-, 2-, 4- and 8-weeks pristane-elicited PECs were subjected to Western blotting using antibodies specific for phosphorylated Akt and total Akt (upper left panel) or antibodies specific for Mcl-1 (upper right panel). In the lower left and right panels the pAkt/Akt ratios and normalized amounts of Mcl-1 are shown, respectively. In all blots, Stain-Free technology was used as the loading control method^88^. Images of the entire non-cropped membranes have been included as supplementary data in Figs S10–S12.

To explore the possibility that altered apoptosis may be responsible for the decreased number of PECs in *Cd38*^−/−^ mice observed at 2- and 4-weeks post pristane treatment, we isolated WT and *Cd38*^−/−^ pristane-elicited PECs and compared their apoptotic phenotype by simultaneously staining these cells with Annexin V-FITC (AnnV) and the cell viability dye 7-aminoactinomycin D (7-AAD) (Fig. 2a). PECs from 1-week pristane-treated WT and *Cd38*^−/−^ mice showed no differences in the numbers of early apoptotic (AnnV^lo^7-AAD^−^), late apoptotic (AnnV^hi^7-AAD^−^) and end-stage apoptotic/dead (AnnV^hi^7-AAD^+^) cells (Fig. 2b, lower left panel). In contrast, PECs from 2-weeks pristane-treated *Cd38*^−/−^ mice had a significant decrease in the number of early apoptotic cells and a modest decrease (*P* = 0.0651) in the number of late apoptotic cells (Fig. 2b, lower right panel).

Normally, cleavage of caspase-3 by caspase-8 or caspase-9 is required for the commitment of cells to apoptosis. The processed form of caspase-3 consists of a large (17 kDa) and a small (12 kDa) subunit, which associate to form the active enzyme. To assess whether cleaved caspase-3 was detected in PECs from pristane-treated mice, we subjected lysates from 2-, 4- and 8-weeks pristane-elicited PECs to Western blotting using an antibody that recognized the 17 kDa subunit of active caspase-3. The amount of cleaved caspase-3 in WT PECs peaked at 2 weeks, and was found to be 4-fold higher relative to *Cd38*^−/−^ PECs at this time point (Fig. 2c, lower left panel).

Cell survival requires the active inhibition of apoptosis, which is accomplished by the phosphorylation of pro-apoptotic factors via the activation of Erk1/2 or Akt, or by promoting the expression of anti-apoptotic factors. We, therefore, measured and compared the levels of phospho-Erk1/2 (pErk), phospho-Akt (pAkt) and the key anti-apoptotic player Mcl-1 in pristane-elicited PECs. WT and *Cd38*^−/−^ PECs reached similar maximal levels of pErk1/2 at 4-weeks post-pristane treatment but differed in phosphorylation kinetics (Fig. 2c, lower right panel). Specifically, WT PECs had slow and sustained ERK1/2 phosphorylation that was low at 2 weeks, reached a peak at 4 weeks and remained maximal at 8 weeks post-pristane treatment. *Cd38*^−/−^ PECs, on the other hand, had rapid and transient ERK1/2 phosphorylation that was 2.2-fold higher at 2 weeks, reached a peak at 4 weeks, and declined 2.5-fold at 8 weeks compared to WT PECs. Comparison of pAkt levels revealed decreased phosphorylation of Akt in *Cd38*^−/−^ PECs at 1-week post-pristane treatment, which was 3.7-fold lower than that in WT PECs (Fig. 2d, lower left panel). The phosphorylation kinetics of Akt, however, did not differ between WT and *Cd38*^−/−^ PECs, and correlated with those of caspase-3. Furthermore, Mcl-1 protein levels were increased in both WT and *Cd38*^−/−^ PECs, with a maximum at 4-weeks post pristane treatment and a kinetic profile very similar to that of pERK1/2 (Fig. 2d, lower right panel). Images of the entire non-cropped membranes from the Western blot analyses have been included as supplementary data in Figs S10–S12.

### *Cd38*^−/−^ and *Trpm2*^−/−^ Ly6C^hi^ monocytes and Ly6C^lo^ monocytes/macrophages exhibit defective pristane-induced apoptosis

**Figure 3:**
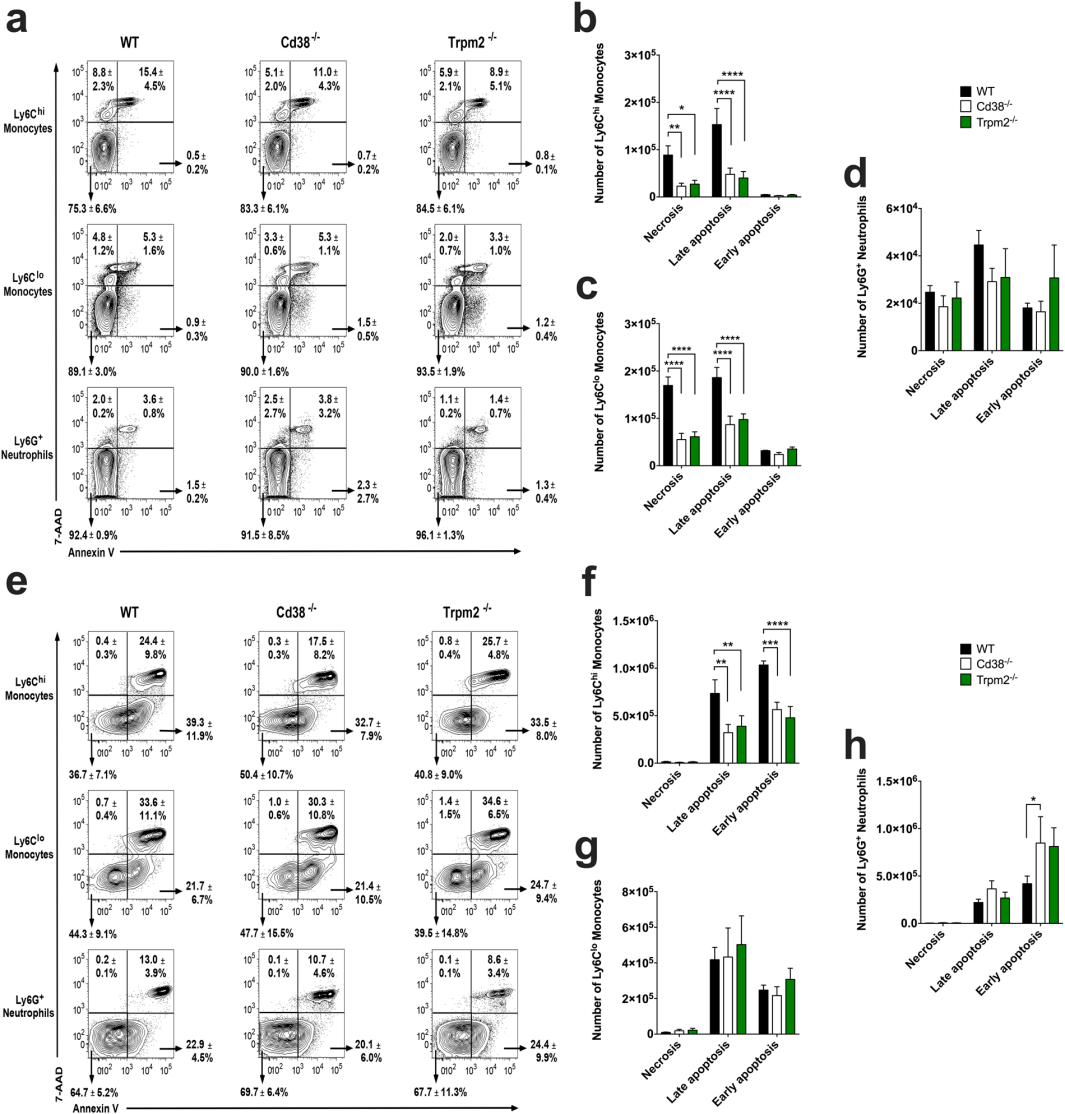
Defective pristane-induced apoptosis in Ly6C^hi^ monocytes and Ly6C^lo^ monocytes/macrophages from *Cd38*^−/−^ and *Trpm2*^−/−^ mice. (**a**,**e**) Flow cytometric analysis of Annexin V (AnnV) and 7-AAD staining of Ly6C^hi^ monocytes, Ly6C^lo^ monocytes/macrophages and Ly6G^+^ neutrophils from 1-week pristane-treated **(a)** and 2-weeks pristane-treated **(e)** WT, *Cd38*^−/−^ and *Trpm2*^−/−^ mice. Frequencies of AnnV^−^7-AAD^−^ live cells, AnnV^+^7-AAD^−^ early apoptotic cells, AnnV^+^7-AAD^+^ end-stage apoptotic/dead cells and AnnV^−^7-AAD^+^ necrotic cells are shown as percentages of the Ly6C^hi^, Ly6C^lo^ and Ly6G^+^ parent cell populations ± SD (n = 5 mice/group). Dot plots shown are representative of 2 independent experiments. **(b–d)** Numbers of early apoptotic, late apoptotic and necrotic Ly6C^hi^ monocytes **(b**), Ly6C^lo^ monocytes/macrophages **(c)** and Ly6G^+^ neutrophils **(d)** from 1-week pristane-treated WT, *Cd38*^−/−^, and *Trmp2*^−/−^ mice. Data are shown as the mean ± SD (n = 5 mice/group). **(f–h)** Numbers of early apoptotic, late apoptotic and necrotic Ly6C^hi^ monocytes **(f)**, Ly6C^lo^ monocytes/macrophages **(g)** and Ly6G^+^ neutrophils **(h)** from 2-weeks pristane-treated WT, *Cd38*^−/−^, and *Trmp2*^−/−^ mice. Data are shown as the mean ± SD (n = 7 mice/group). *P < 0.05 **P < 0.01 ***P < 0.001 ****P < 0.0001 by 2-way ANOVA with the Dunnett’s multiple comparisons test.

To examine whether there was a specific peritoneal cell subset in *Cd38*^−/−^ or TRPM2-deficient (*Trpm2*^−/−^) mice with defective pristane-induced and apoptosis-mediated cell death, we isolated WT, *Cd38*^−/−^ and *Trpm2*^−/−^ PECs from 1- and 2-weeks pristane-treated mice and assessed early apoptotic (AnnV^+^7-AAD^−^), late apoptotic (AnnV^+^7-AAD^+^) and necrotic (AnnV^−^7-ADD^+^) cells within 3 major cell populations, namely Ly6C^hi^ monocytes, Ly6C^lo^ monocytes/macrophages, and Ly6G^+^ neutrophils (Fig. 3a and e). At 1-week post-pristane treatment, *Cd38*^−/−^ and *Trpm2*^−/−^ mice had decreased numbers of necrotic and late apoptotic Ly6C^hi^ monocytes and Ly6C^lo^ monocytes/macrophages compared to WT mice (Fig. 3b and c), while the numbers of necrotic/apoptotic Ly6G^+^ neutrophils were similar (Fig. 3d). The Ly6C^lo^ cell population was further characterized as 2 distinct subsets based on side scatter (SSC), which is indicative of granularity, and identified accordingly as Ly6C^lo^SSC^hi^ cells (macrophages) and Ly6C^lo^SSC^lo^ cells (monocytes) (Fig. S1A). Interestingly, the defective apoptosis/necrosis exhibited by *Cd38*^−/−^ and *Trpm2*^−/−^ Ly6C^lo^ cells at 1-week post treatment corresponded to that of the SSC^hi^ subset (Fig. S1B), which comprised the majority of the CD11b^+^ cells (Fig. S1A). A similar defective apoptotic phenotype in *Cd38*^−/−^ and *Trpm2*^−/−^ Ly6C^hi^ monocytes was also observed at 2-weeks post pristane treatment (Fig. 3f). Indeed, *Cd38*^−/−^ and *Trpm2*^−/−^ mice had significantly lower numbers of early- and late-apoptotic Ly6C^hi^ monocytes compared to WT mice. No differences were detected in apoptotic Ly6C^lo^ monocytes/macrophages between the 3 groups of mice at this time point (Fig. 3g), but a modest increase in early-apoptotic *Cd38*^−/−^ and *Trpm2*^−/−^ Ly6G^+^ neutrophils was observed (Fig. 3h). These data suggest that pristane-induced apoptosis/necrosis of Ly6C^hi^ monocytes and Ly6C^lo^ monocytes/macrophages is both CD38- and TRPM2-dependent. This link between CD38 and TRPM2 is also supported by additional phenotypic similarities between pristane-treated *Cd38*^−/−^ and *Trpm2*^−/−^ mice, specifically their decreased numbers of total PECs, and decreased frequency and numbers of Ly6C^hi^ monocytes recruited to the PC (Fig. S2). Interestingly, at 2-weeks post-pristane treatment, *Art2*^−/−^ mice showed a higher frequency of apoptotic Ly6C^hi^ monocytes and Ly6C^lo^ monocytes/macrophages compared to *Cd38*^−/−^ and *Cd38*^−/−^*Art2*^−/−^ double knockout mice (Fig. S3), providing further evidence that ART2 does not play a role in this process.

### Distinct clustering of mice based on ISG expression in pristane-elicited PECs

**Figure 4:**
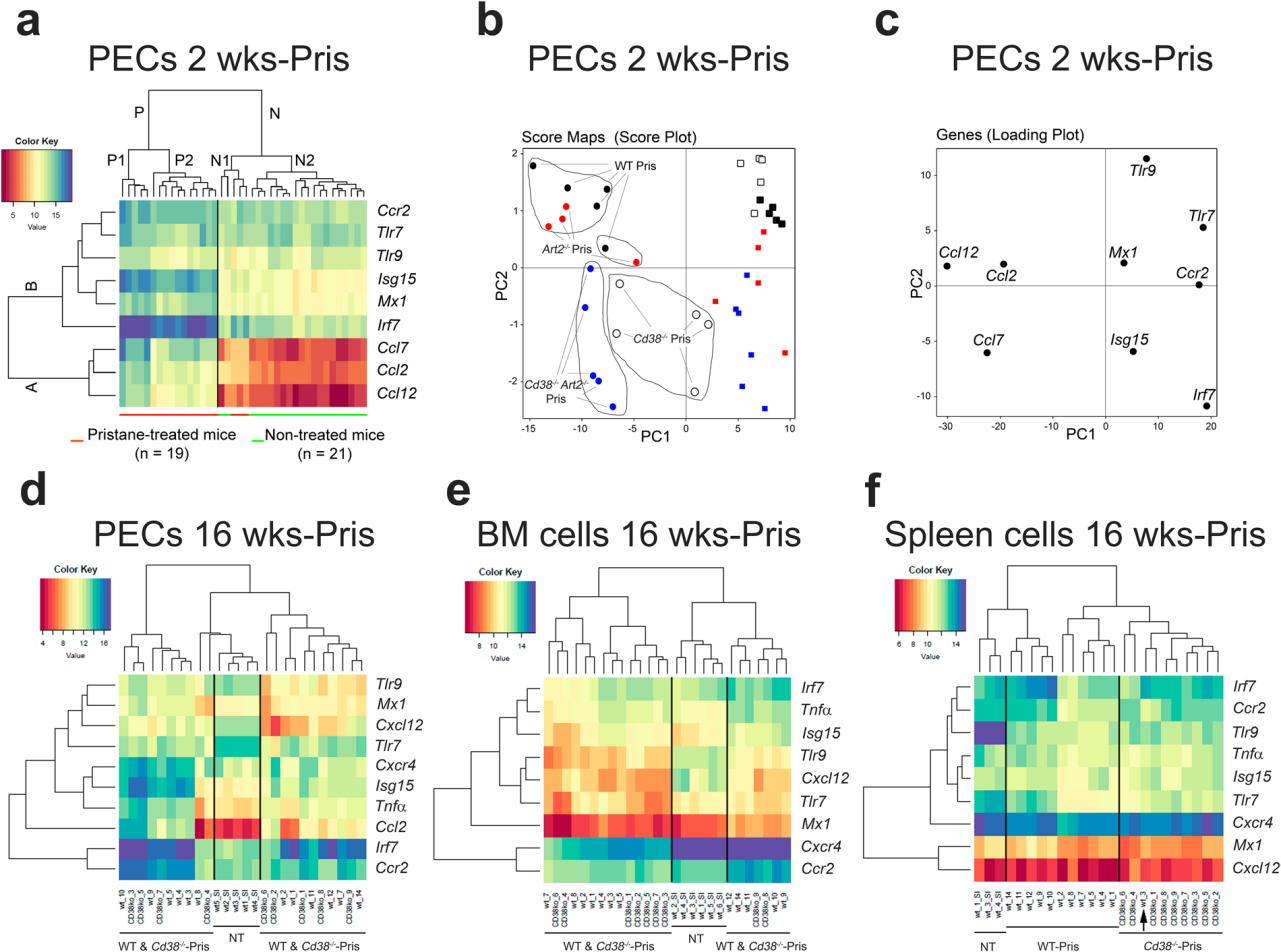
Distinct clustering of mice according to the expression of Type I IFN-inducible genes in pristane-treated vs. non-treated PECs. (**a**) Heat-map analysis of 9 genes, namely *Ccr2*, *Tlr7*, *Tlr9*, *Isg15*, *Mx1*, *Irf7*, *Ccl7*, *Ccl12* and *Ccl12*, whose expression was useful in discriminating 2-weeks pristane-treated (cluster P) from control non-treated WT mice (cluster N). Gene expression profiles were separated into 2 major clusters (A and B), and each of them was further sub-dived into minor clusters according to expression pattern. (**b**) Principal Component Analysis (PCA) on the 9 selected genes showing a distinct expression profile in 2-weeks pristane-treated vs. control non-treated mice. The score plot shows a spot map of the 40 mice tested (black circles: pristane-treated WT; black squares: non-treated WT; open circles: pristane-treated *Cd38*^−/−^; open squares: non-treated *Cd38*^−/−^; red circles: pristane-treated *Art2*^−/−^; red squares: non-treated *Art2*^−/−^; blue circles: pristane-treated *Cd38*^−/−^*Art2*^−/−^; blue squares: non-treated *Cd38*^−/−^*Art2*^−/−^) projected onto the first two principal components. The drawing lines highlight the clustering of mice of the same genetic background in distinct subareas according to their distinct gene expression profile in response to pristane treatment. (**c**) Loading plot of the 9 selected genes with expression levels projected onto the first two principal components. The plot shows that the genes clustered in distinct quadrants according to their differential expression in the 8 groups of experimental mice tested. (**d–f**) Heat-map analyses of gene expression in PECs (**d**) BM cells (**e**) and spleen cells (**f**) from 16-weeks pristane-treated WT and *Cd38*^−/−^ mice compared to their non-treated WT counterpart.

We measured and compared the expression of ISGs in PECs from 2-weeks pristane-treated WT, *Cd38*^−/−^, *Art2*^−/−^, and *Cd38*^−/−^*Art2*^−/−^ mice, and observed a CD38-dependent, but ART2-independent, pristane-induced ISG expression profile (Fig. S4). To determine whether we could discriminate the groups of mice based on their ISG expression profile, unsupervised hierarchical clustering of gene expression was performed. We identified two distinct groups, clusters P and N, which clearly distinguished between pristane-treated and non-treated mice, respectively (Fig. 4a). Gene expression was grouped into 2 major clusters, A and B (Fig. 4a). Cluster A comprised of the 3 chemokine genes, namely *Ccl7*, *Ccl2* and *Ccl12*, which showed the major differences in expression between pristane-treated and non-treated mice, while cluster B included the ISGs (*Irf7*, *Isg15*, and *Mx1)* as well as *Tlr9*, *Ccr2* and *Tlr7* that were differently distributed in subclusters. We used Principal Component analysis (PCA), a multivariate statistical approach, to confirm and select the best candidate genes for distinguishing pristane-treated from non-treated groups of mice. Figure 4b shows a score plot of the distribution of 40 mice according to the expression variance of the 9 genes tested. Pristane-treated *Art2*^−/−^ mice clustered in the upper left quadrant together with pristane-treated WT mice, indicating limited variance between these two groups of mice. The pristane-treated *Cd38*^−/−^*Art2*^−/−^ double knockout mice clustered exclusively in the lower left quadrant, while the pristane-treated *Cd38*^−/−^ mice clustered in the two lower quadrants in an intermediate area located between pristane-treated mice to the left and non-treated mice to the right. The non-treated WT and *Cd38*^−/−^ mice clustered in the upper right panel (black and open squares, respectively), while most of the non-treated *Art2*^−/−^ mice, and all of the non-treated *Cd38*^−/−^ *Art2*^−/−^ double knockout mice clustered in the lower right panel (red and blue squares, respectively). In summary, mice clustered in different subareas depending on whether they were treated with pristane and whether they differed in CD38 expression. We generated a gene loading plot to cluster each individual gene in an attempt to identify strain-specific gene expression profiles that could be used to distinguish the groups of mice analysed (Fig. 4c). The expression of chemokines *Ccl2* and *Ccl12*, which clustered in the upper left quadrant, associated with pristane-treated WT and *Art2*^−/−^ mice, while the expression of chemokine *Ccl7*, which clustered in the lower left quadrant, associated with pristane-treated *Cd38*^−/−^ and *Cd38*^−/−^*Art2*^−/−^ mice. The expression of the other genes tested, namely *Irf7*, *Isg15*, *Mx1*, *Tlr9*, *Ccr2* and *Tlr7*, clustered in the right quadrants and therefore associated with the non-treated groups of mice. The similar clustering into the upper right quadrant of non-treated WT and *Cd38*^−/−^ mice (score plot in Fig. 4b) and the *Tlr7*, *Tlr9*, and *Mx1* genes (loading plot in Fig. 4c) suggests that the expression profile of these genes could be used to discriminate non-treated WT and *Cd38*^−/−^ mice from the other groups of mice analysed.

### Expression of ISGs and TNF-α-regulated genes in splenic cells discriminate between 16-weeks pristane-treated *Cd38*^−/−^ and WT mice

We also performed unsupervised hierarchical clustering of gene expression in PECs, BM, and splenic cells from 16-weeks pristane-treated WT and *Cd38*^−/−^ mice. Since i.p. injection of pristane induces BM abnormalities and suppresses BM *Cxcl12* mRNA expression by a TLR7-driven and TNF-α-mediated mechanism, which is IFN-I-independent^29^, we included gene expression analyses of *Tnfα*, *Cxcl12* and the CXCL12 receptor *Cxcr4*. Despite the expected pristane-elicited decreased expression of *Cxcl12* and increased expression of *Tnfα* in WT BM cells, no differences were observed in the expression of these genes between pristane-elicited WT and *Cd38*^−/−^ BM cells (Fig. S5), with the exception of *Cxcr4* that was overexpressed, and *Ccl12* and *Irf7* that were underexpressed in *Cd38*^−/−^ BM cells (Fig. S5).

Unsupervised hierarchical clustering of gene expression in PECs, BM and splenic cells from 16-weeks pristane-treated WT and *Cd38*^−/−^ mice identified distinct clusters of non-treated and pristane-treated mice (Fig. 4d–f). Interestingly, pristane-elicited splenic cells could be separated into 2 subclusters, one consisting exclusively of WT mice, and the other mostly of *Cd38*^−/−^ mice (Fig. 4f). Conversely, no clear separation could be achieved between pristane-elicited WT and *Cd38*^−/−^ PECs (Fig. 4d) and BM cells (Fig. 4e). These results suggest that the differences in gene expression profiles between pristane-treated WT and *Cd38*^−/−^ mice shifted from the PC and the BM at the 2-weeks time point to secondary lymphoid tissues, such as the spleen, by the 16-weeks time point.

### Decreased CCL2 and IL-1α protein levels in the peritoneal lavage fluid of pristane-treated *Cd38*^−/−^ mice

**Figure 5:**
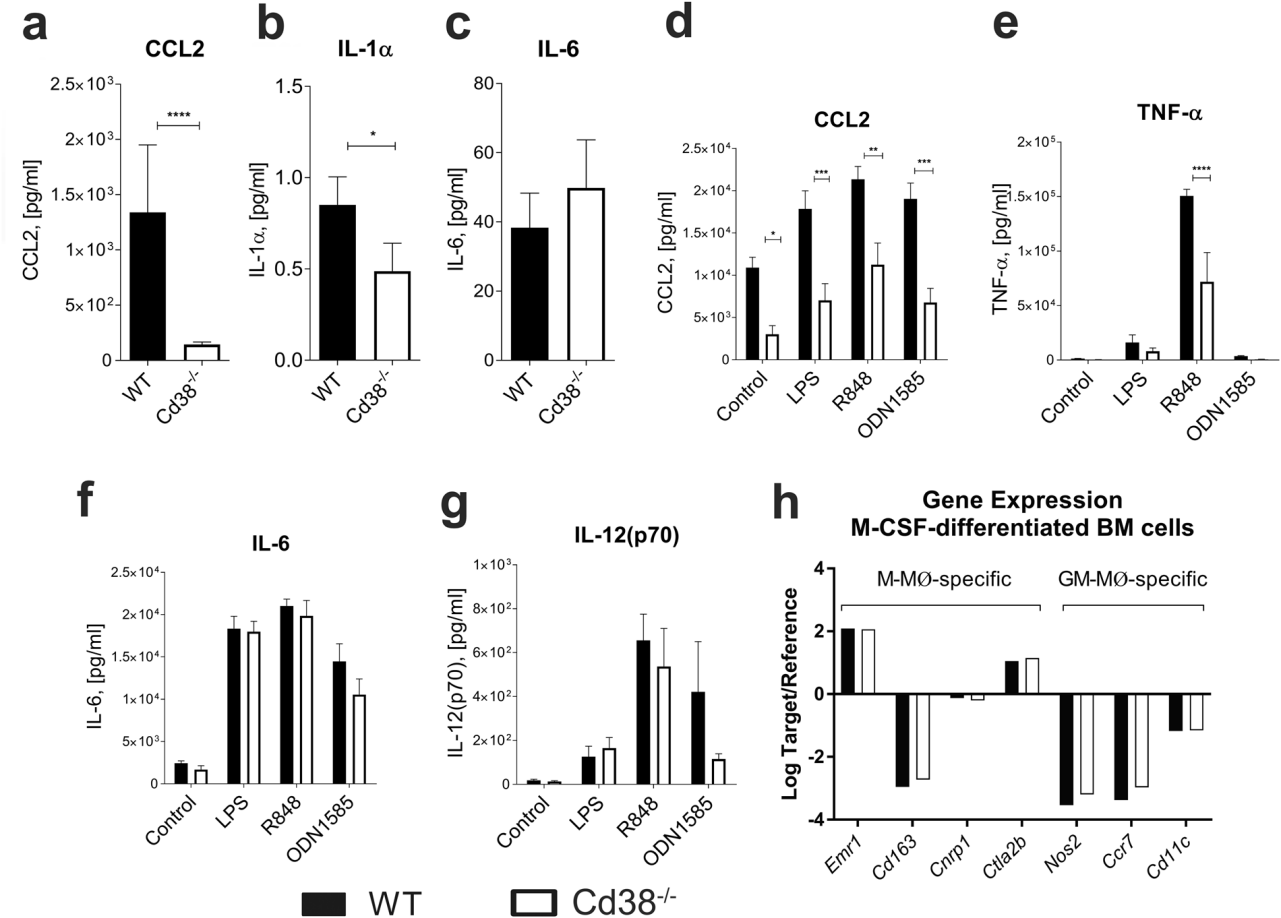
Defective pro-inflammatory phenotype of *Cd38*^−/−^ PECs but not BM-derived macrophages. (**a–c**) Decreased *in vivo* secretion of CCL2 and IL-1α in pristane-treated *Cd38*^−/−^ mice. Concentration of CCL2 **(a)**, IL-1α **(b)** and IL-6 **(c**) proteins (pg/ml) in the peritoneal lavage fluid of 2-weeks pristane-treated WT and *Cd38*^−/−^ mice. Data are shown as the mean ± SE and represent cumulative data from 3 independent experiments (n = 15–22/group). *P < 0.05 ****P < 0.0001 by the Mann Whitney test. (**d–g**) Decreased *in vitro* secretion of CCL2 and TNF-α by pristane-elicited *Cd38*^−/−^ PECs in in response to the TLR7 agonist R848. Concentration of CCL2 **(d)**, TNF-α **(e)**, IL-6 **(f)**, and IL-12(p70) **(g)** proteins (pg/ml) in the supernatants of 2-weeks pristane-elicited PECs from WT and *Cd38*^−/−^ mice (5 mice/group) stimulated *in vitro* for 24 hours at 37 °C and 5% CO_2_ with LPS, R848 or ODN1585. Each condition was tested in triplicate wells. Data are shown as the mean ± SE. *P < 0.05 **P < 0.01 ***P < 0.001 by 2-way ANOVA with Bonferroni’s multiple comparisons test. **(h)** Normal M-CSF-induced differentiation of *Cd38*^−/−^ BM cells into anti-inflammatory macrophages. BM cells were isolated from naïve WT and *Cd38*^−/−^ mice and differentiated *in vitro* into anti-inflammatory macrophages (M-MØ) by culturing them for 7 days in the presence of murine M-CSF as described in Methods. Gene expression of mouse M-MØ-specific gene markers *Emr1*, *Cd163*, *Cnrp1*, and *Ctla2b*, and pro-inflammatory macrophage (GM-MØ)-specific gene markers *Nos2*, *Ccr7*, and *Cd11c* was measured by Q-PCR.

We measured the protein levels of the IFN-I-inducible chemokine CCL2 in the peritoneal lavage fluid, and found them to be significantly lower in 2-weeks pristane-treated *Cd38*^−/−^ mice compared to WT mice (Fig. 5a). Moreover, the concentration of IL-1α, which is involved in neutrophil recruitment^30^, was significantly diminished in *Cd38*^−/−^ mice (Fig. 5b). In contrast, the protein levels of the pro-inflammatory cytokine IL-6 were similar in both mice (Fig. 5c), while those of TNF-α and IL-12 were undetectable (data not shown). Next, we analysed the expression of CCR2, which is the major receptor for chemokines CCL2, CCL7 and CCL12, in different PEC populations from 2-weeks pristane-treated WT and *Cd38*^−/−^ mice (Fig. S6). The number of CCR2^hi^Ly6C^hi^ monocytes was significantly lower in *Cd38*^−/−^ mice compared to WT mice, while the number of CCR2^+^Ly6G^+^ neutrophils and CCR2^+^Ly6C^lo^ monocytes were similar between the two groups of mice (Fig. S6). Indeed, these results are in agreement with the lower levels of CCL2 detected in the peritoneal lavage fluid from pristane-treated *Cd38*^−/−^ mice (Fig. 5a).

### Defective secretion of CCL2 and TNF-α by pristane-elicited PECs from *Cd38*^−/−^ mice following TLR7 agonist stimulation

**Figure 6:**
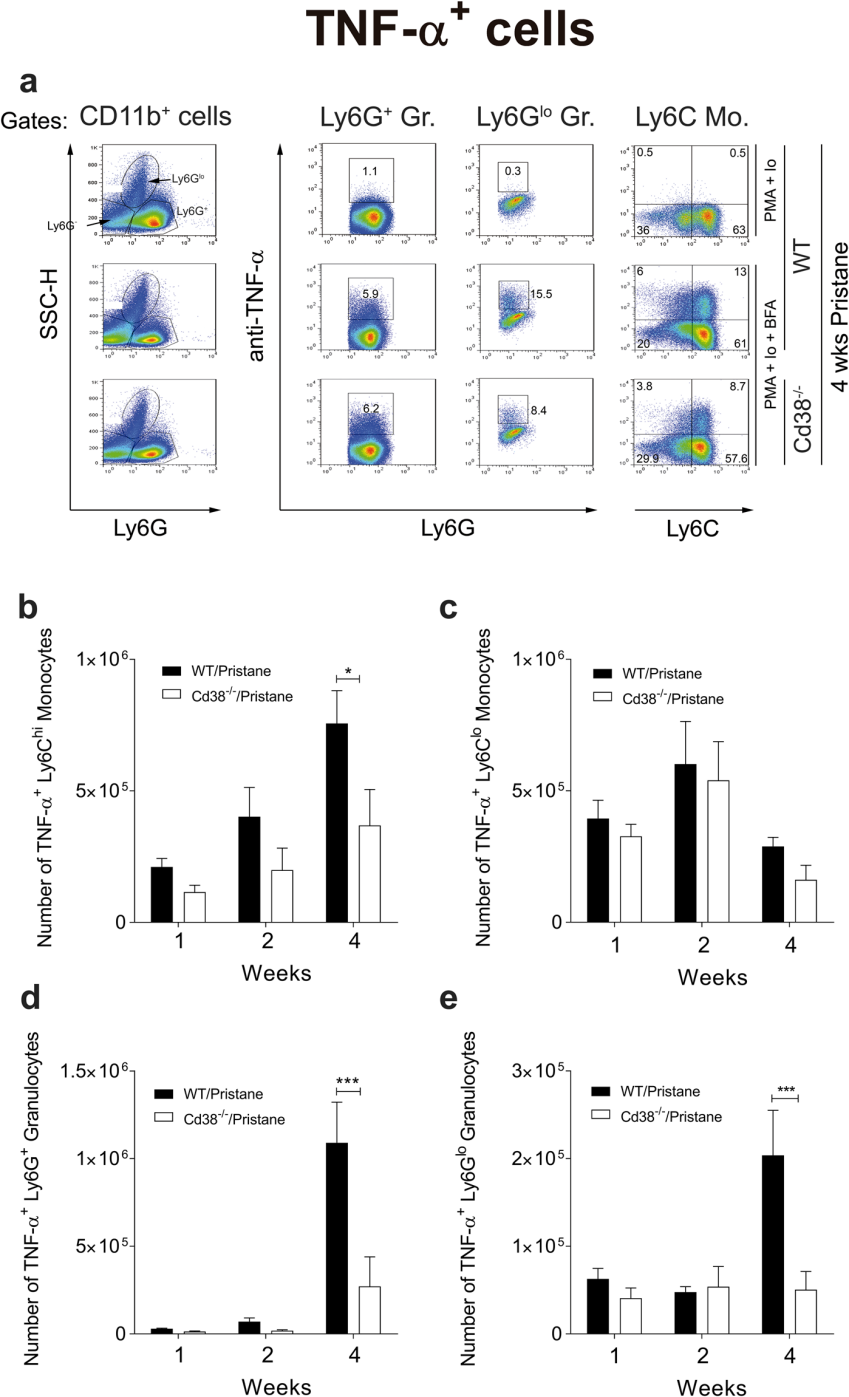
Defective recruitment of TNF-α-producing neutrophils and Ly6C^hi^ monocytes to the peritoneum of pristane-treated *Cd38*^−/−^ mice. (**a**) Flow cytometric analyses of 4-weeks pristane-elicited PECs from WT and *Cd38*^−/−^ mice first surface-stained with anti-CD11b, anti-Ly6G and anti-Ly6C fluorescent-labelled antibodies, and then intracellularly stained for TNF-α as described in Methods. The three CD11b^+^ peritoneal cell populations of interest were identified based on their level of Ly6G expression and side scatter (SSC) characteristics. The cell types were SSC^lo^Ly6G^+^ granulocytes (neutrophils), and SSC^hi^Ly6G^lo^ granulocytes (eosinophils) and SSC^lo^Ly6G^−^ monocytes. Each cell type was stimulated with PMA, ionomycin and Brefeldin A (BFA). As a control, each WT cell type was incubated with PMA and ionomycin alone. The level of TNF-α expression in each cell type is shown, and the representative frequencies are included in each gate. (**b–e**) Numbers of TNF-α^+^ Ly6C^hi^ monocytes (**b**), TNF-α^+^ Ly6C^lo^ monocytes (**c**), TNF-α^+^ Ly6G^+^ granulocytes/neutrophils (**d**), and TNFα^+^ Ly6G^lo^ granulocytes/eosinophils (**e**) at 1-, 2- and 4-weeks post pristane treatment. Data are shown as the mean ± SE (n = 6–7 mice/group). All P values were determined by 2-way ANOVA with the Sidak’s multiple comparisons test. *P < 0.05 **P < 0.01 ***P < 0.001.

We next assessed the functional capacity of pristane-elicited PECs to respond to several TLR agonists. PECs from 2-weeks pristane-treated *Cd38*^−/−^ mice produced significantly less CCL2 in response to *in vitro* stimulation with LPS (TLR4 agonist), R848 (TLR7 agonist), or ODN1585 (TLR9 agonist) (Fig. 5d). Interestingly, non-stimulated control *Cd38*^−/−^ PECs showed decreased secretion of CCL2 as well (Fig. 5d). Similarly, *Cd38*^−/−^ PECs produced less TNF-α in response to R848 compared to WT PECs (Fig. 5e). In contrast, no differences in the secretion of IL-6 and IL12(p70) were observed between WT and *Cd38*^−/−^ PECs in response to these stimuli (Figs 5f and 6g, respectively). To determine whether the impaired TLR-mediated secretion of CCL2 and TNF-α by *Cd38*^−/−^ PECs was due to intrinsic TLR signalling defects, we sorted peritoneal Ly6C^hi^ monocytes, Ly6C^lo^ monocytes/macrophages and Ly6G^+^ neutrophils from 2-weeks pristane-treated WT and *Cd38*^−/−^ mice and assessed their gene expression profiles. The sorting strategy used to purify these cell populations is shown in Fig. S7. Gene expression analyses (Fig. S8) and *in vitro* TLR agonist stimulations (data not shown) revealed no major intrinsic TLR signalling defects in *Cd38*^−/−^ Ly6C^hi^ monocytes, Ly6C^lo^ monocytes/macrophages and Ly6G^+^ neutrophils. Interestingly, *Ccl2* gene expression was significantly upregulated in *Cd38*^−/−^ Ly6C^hi^ monocytes, indicative of a transcriptional effort by these cells to compensate for their impaired CCL2 protein secretion.

### CD38 deficiency does not impair M-CSF-induced differentiation of BM cells into anti-inflammatory macrophages

Increased *Cd38* gene expression has been reported in human anti-inflammatory M-CSF-dependent macrophages (M-MØ) compared to pro-inflammatory GM-CSF-dependent macrophages (GM-MØ)^31^ (Gene Expression Ommnibus reference: GSE68061). To assess whether the absence of CD38 affected the polarization of BM-derived macrophages, we isolated BM cells from *Cd38*^−/−^ and WT naïve mice and differentiated them *in vitro* into anti-inflammatory macrophages as previously described^32^. Subsequent analysis of the mouse M-MØ-specific gene markers *Emr1*, *Cd163*, *Cnrp1*, and *Ctla2*, and the GM-MØ-specific gene markers *Nos2*, *Ccr7*, and *Cd11c* revealed that there were no significant differences in the expression of these genes between *Cd38*^−/−^ and WT cells (Fig. 5h), suggesting that CD38 does not play a role in the polarization of BM macrophages towards an anti-inflammatory phenotype. Interestingly, M-CSF-stimulated *Cd38*^−/−^ BM cells manifested enhanced expansion compared to WT cells, as evidenced by the greater number of recovered cells after 7 days in culture (1.85-fold increase, data not shown). Whether CD38 modulates M-CSF-induced proliferation of BM cells^33,34^ requires further investigation.

### Defective recruitment of TNF-α-producing Ly6G^+^ neutrophils and Ly6C^hi^ monocytes to the peritoneum of pristane-treated *Cd38*^−/−^ mice

To further assess TNF-α production in *Cd38*^−/−^ mice, we harvested PECs from mice at 1-, 2-, and 4-weeks post-pristane treatment and cultured them *in vitro* with Brefeldin A to inhibit protein transport and enhance intracellular TNF-α staining for flow cytometric detection. TNF-α was produced almost exclusively by CD11b^+^ cells, specifically by Ly6G^−^ Ly6C^+^ monocytes and by Ly6C^−^ Ly6G^+^ granulocytes (Fig. 6a). Two subpopulations of Ly6G^+^ cells were characterised based on granularity, despite differences in SSC properties between fixed/permeabilised and non-manipulated cells that made the nature of the subpopulations uncertain. However, since neutrophils are the most abundant granulocytes in pristane-treated mice, and eosinophils are Ly6G^int-neg^SSC^hi^ granulocytes^35^, we designated Ly6G^+^SSC^lo^ and Ly6G^lo^SSC^hi^ cells as neutrophils and eosinophils, respectively (Fig. 6a). In agreement with previously published reports, we observed an increase in the number of pristane-elicited TNF-α^+^Ly6G^+^ neutrophils in WT mice over time^29,36^, which outnumbered the other cell types analysed (Fig. 6d). Importantly, at 4-weeks post-pristane treatment, which is the peak of the acute inflammatory response, *Cd38*^−/−^ mice had decreased numbers of TNF-α^+^Ly6C^hi^ monocytes (Fig. 6b), Ly6G^+^ neutrophils (Fig. 6d) and Ly6G^lo^ eosinophils (Fig. 6e) compared to WT mice. No differences were observed in the number of TNF-α^+^Ly6C^lo^ monocytes between *Cd38*^−/−^ and WT mice (Fig. 6c).

To investigate whether the defective recruitment of TNF-α-producing Ly6G^+^ neutrophils and Ly6C^hi^ monocytes to the peritoneum of pristane-treated *Cd38*^−/−^ mice was due to intrinsic chemotactic defects, we assessed the chemotaxis of mouse BM monocytes to CCL2 and CXCL12 *in vitro* (Fig. S9a,b) and the migration of Ly6G^+^ neutrophils to the peritoneum in response to *in vivo* zymosan challenge (Fig. S9c,d). Similar responses between WT and *Cd38*^−/−^ mice were observed, suggesting that the migration of these cells in response to pristane was CD38-independent.

### Decreased autoantibody production and renal inflammation in *Cd38*^−/−^ mice

**Figure 7:**
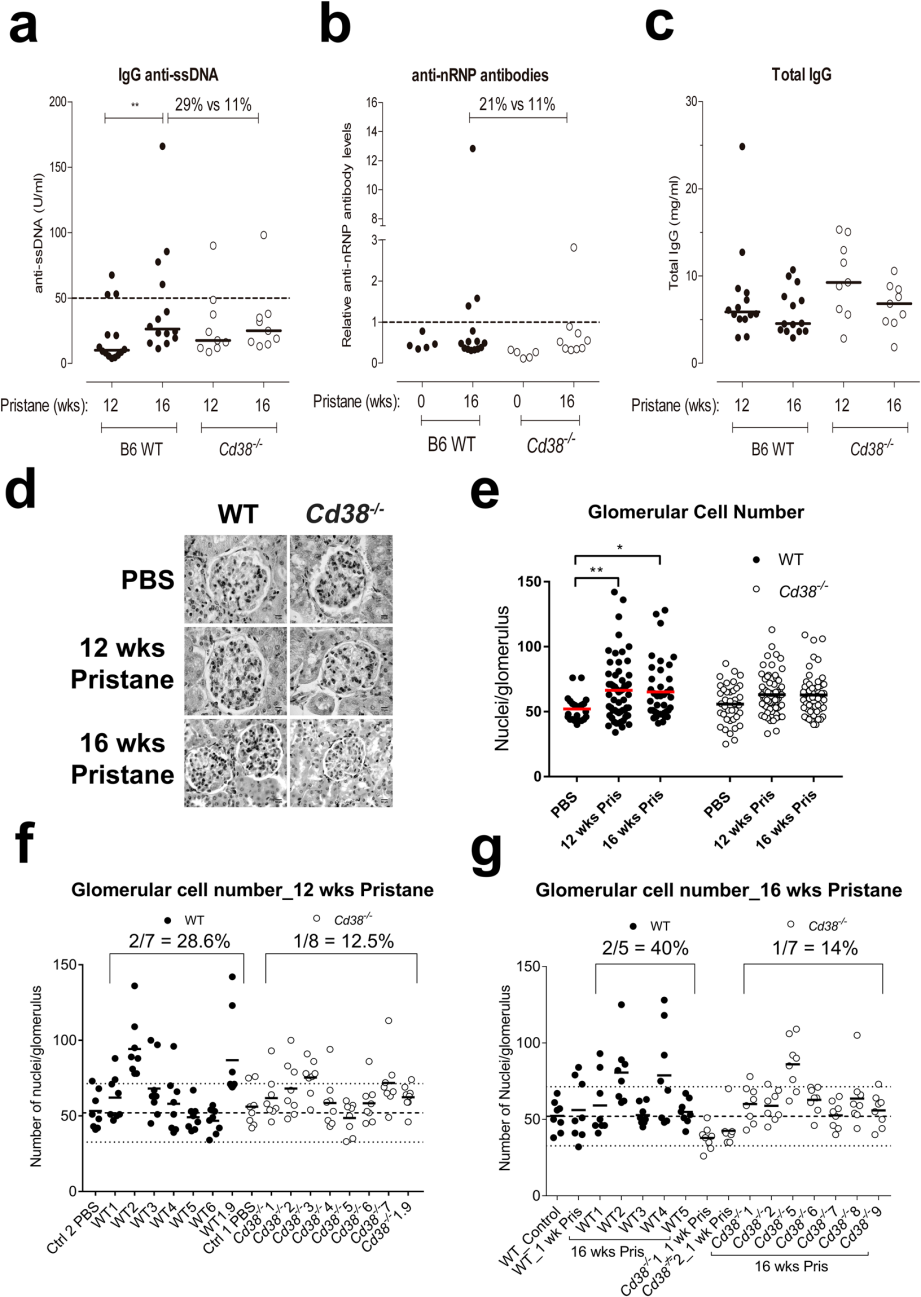
Diminished autoantibody production and attenuated glomerulonephritis in pristane-treated *Cd38*^−/−^ mice. (**a**) IgG anti-single-stranded DNA (ssDNA) autoantibody serum levels in *Cd38*^−/−^ mice (n = 9) and WT control mice (n = 14) at 12- and 16-weeks post pristane treatment. Dashed line indicates the concentration (U/ml) of anti-ssDNA antibodies above which the anti-ssDNA levels were considered statistically significant. (**b**) Anti-nRNP autoantibody levels in non-treated and 16-weeks pristane-treated *Cd38*^−/−^ and WT mice. Dashed line indicates the normalized value of anti-nRNP antibody levels calculated as described in Methods. (**c**) Serum levels of total IgG (mg/ml) in 12- and 16-weeks pristane-treated *Cd38*^−/−^ and WT mice. Horizontal bars represent the mean value for each group. (**d**) H&E stained sections of kidneys from WT and *Cd38*^−/−^ mice 12-weeks post PBS or pristane treatment, and 16-weeks post pristane treatment. Original magnification × 100. In each panel horizontal bars indicate the distance in μm. (**e**) Cumulative data of glomerular cell numbers in WT (n = 3) and *Cd38*^−/−^ (n = 5) mice 12-weeks post PBS treatment, WT (n = 7) and *Cd38*^−/−^ (n = 8) mice 12-weeks post pristane treatment, and WT (n = 5) and *Cd38*^−/−^ (n = 7) mice 16-weeks post pristane treatment. Glomerular cellularity was evaluated by counting the number of nuclei per glomerular cross-section (8 glomeruli/mouse). *P < 0.05 **P < 0.01 by 2-way ANOVA with Bonferroni’s multiple comparisons test. (**f**,**g**) Glomerular cellularity of each individual mouse at 12-weeks **(f)** and 16-weeks **(g)** post pristane treatment. Dashed lines represent the mean value for the number of nuclei per glomerulus in PBS-treated WT mice, and the dotted lines represent the upper and lower limits of normality (mean ± 2 SD).

Pristane does not induce IgG anti-dsDNA antibodies in B6 mice, however it induces anti-ssDNA antibodies^37^. Hence, we tested anti-ssDNA antibodies at 12- and 16-weeks post-pristane treatment to determine whether lack of CD38 was protective. WT mice exhibited a significant increase in IgG anti-ssDNA antibody serum levels at 16-weeks compared to 12-weeks post-pristane (median values: 26.3 U/ml at 16-weeks vs. 10.0 U/ml at 12-weeks, *P* = 0.0082, Mann Whitney test, n = 14) (Fig. 7a). *Cd38*^−/−^ mice, on the other hand, had no differences between the two time points (median values: 24.9 U/ml at 16-weeks vs. 17.6 U/ml at 12-weeks, *P* = 0.1387, Mann Whitney test, n = 9). Furthermore, significantly fewer pristane-treated *Cd38*^−/−^ mice (11%, n = 9) were positive for anti-nRNP autoantibodies compared to WT mice (21%, n = 14) (Fig. 7b). The frequency of anti-nRNP antibodies we observed in pristane-treated WT mice was consistent with previously published values (24% of pristane-treated female B6 mice)^28^. In both *Cd38*^−/−^ and WT mice, the serum levels of total IgG were lower at 16-weeks compared to 12-weeks post-pristane treatment (Fig. 7c).

We next examined the effect of CD38 deficiency on the induction of lupus nephritis by evaluating glomerular cellularity at 12- and 16-weeks post-pristane treatment. Pristane-treated WT mice had increased glomerular cellularity compared to saline-treated control mice at both time points analyzed (Fig. 7d and e). Pristane-treated *Cd38*^−/−^ mice, on the other hand, showed no statistically-significant difference in glomerular cellularity compared to saline-treated *Cd38*^−/−^ control mice. The normal/background range of glomerular cellularity was calculated by analysing each individual saline-treated WT mouse, and found to be 71.4-32.7 (mean ± 2 SD of the number of nuclei/glomerulus). The percentage of WT mice with glomerular cellularity above the background 71.4 value was significantly higher at 16-weeks compared to 12-weeks post-pristane (40% vs. 28.6%), whereas the percentages of *Cd38*^−/−^ mice remained constant at 13-14% (Fig. 7f and g). Therefore, the relatively mild renal disease induced by pristane in B6 WT mice^37^ was attenuated in *Cd38*^−/−^ mice.

## Discussion

CD38 deficiency results in decreased accumulation of Ly6C^hi^ monocytes, Ly6C^lo^ monocytes/macrophages and Ly6G^+^ neutrophils, and attenuated inflammation at 2- and 4-weeks post pristane treatment. Overall, our results imply that CD38 contributes to the development of pristane-induced lupus, particularly during the inflammatory phase prior to the onset of the autoimmune phase of the disease, which occurs 12–16 weeks post treatment. This is consistent with other models of inflammatory disorders in which *Cd38*^−/−^ mice show only mild disease manifestations, namely CIA^21^, focal cerebral ischemia^38^, allergen-induced airway hyperresponsiveness^39^ and intestinal inflammation/colitis^40^. In these models, attenuated inflammation results from reduced production of cytokines and chemokines, as well as reduced migration of inflammatory cells. In this study, the attenuation of inflammation in *Cd38*^−/−^ mice occurred at the peak of the inflammatory phase (2–4 weeks). Interestingly, during the early inflammatory phase (1-week), no attenuation was observed in the initial recruitment of Ly6C^hi^ monocytes and Ly6G^+^ neutrophils to the peritoneum, and there was no defective production of pro-inflammatory cytokines/chemokines required for the recruitment of these cells to the peritoneum (data not shown). Furthermore, there was no impairment of the *in vitro* chemotactic response of *Cd38*^−/−^ BM-derived Ly6C^hi^ monocytes to CCL2 and CXCL12, or the *in vivo* migratory capacity of Ly6G^+^ neutrophils to respond to an acute zymosan challenge in *Cd38*^−/−^ mice. These data suggest that there is no intrinsic defect in the migration of these Cd38^−/−^ cells from the BM to the peritoneum in response to pristane.

Pristane arrests cell growth in murine peritoneal cells and induces cell death by apoptosis via the mitochondrial pathway of caspase activation^41^. Pyroptosis, defined as caspase-1-dependent cell death following inflammasome activation, is also operative in pristane-treated mice^42^. Our data show that injection of pristane into the peritoneum induces apoptosis-mediated cell death of PECs, as evidenced by the increased number of annexin V^+^ peritoneal cells and their increased levels of cleaved/active caspase-3. Indeed, the higher levels of activated caspase-3 protein in WT PECs, particularly at 2-weeks post pristane treatment, are indicative of a higher rate of apoptosis compared to *Cd38*^−/−^ cells. In contrast, no differences were observed in the levels of MCL-1, an anti-apoptotic protein and member of the BCL2 family^43^. Furthermore, kinases ERK1/2 and AKT showed distinct activation kinetics in pristane-elicited PECs. While caspase-3 activation followed similar kinetics to AKT activation in both WT and *Cd38*^−/−^ PECs, ERK activation correlated with increased levels of MCL-1. MCL-1 is crucial for the survival of neutrophils, and there are many signalling pathways that contribute to its stabilization and expression, including AKT, ERK and agonists of TLR4, TLR7 and TLR9, either via direct phosphorylation or indirectly via NF-κB activation^44^. The persistent activation of ERK in WT PECs, which was not observed in *Cd38*^−/−^ PECs, may also account for the stronger pro-inflammatory response of WT PECs to pristane challenge. Recent studies have shown that in addition to its role as a survival factor and tumor promoting agent, AKT is also able to exhibit pro-apoptotic effects under diverse conditions, including oxidative stress^45–47^, cytokine stimulation^48,49^ and exposure to cytotoxic chemicals like staurosporine, methotrexate, docetaxel and etoposide^49,50^. Moreover, phosphorylation of second mitochondria-derived activator of caspases (SMAC) by AKT promotes caspase-3 activation during etoposide-induced apoptosis in HeLa cells^51^. Our data are in agreement with these studies, and demonstrate that in some instances AKT activation is associated with apoptotic signals and not with survival signals. Further studies, however, are required to identify specific pro- and anti-apoptotic target proteins that are phosphorylated by ERK or AKT following pristane treatment, and that regulate the apoptotic process.

There is evidence that enzymatic activity of CD38 is important for regulating apoptosis of immune cells. NAD is converted by CD38 into ADPR that can bind to and activate the Ca^2+^-permeable channel TRPM2^52^. CD38 also produces other TRPM2 regulators, namely cADPR and NAADP, which can synergize with ADPR to potentiate TRPM2 activation^10^. TRPM2 is highly expressed in phagocytes and since the influx of Ca^2+^ through TRPM2 results in activation of extrinsic and intrinsic cell death pathways^10,53–56^ it is probable that the NAD-ADPR-TRPM2 pathway participates in the regulation of pristane-induced apoptosis. We show that pristane-induced apoptosis is similarly defective in *Trpm2*^−/−^ and *Cd38*^−/−^ Ly6C^hi^ monocytes. This suggests that CD38- and ADPR/TRPM2-mediated calcium signalling plays an important role in pristane-induced apoptosis of peritoneal monocytes. ADPR can be generated either by the interplay between poly(ADP-ribose) polymerases (PARPs) and their catabolic counterpart poly(ADP-ribose) glycohydrolase (PARG)^57,58^ or by CD38^59,60^. CD38 is generally thought of as a type II membrane-bound ectoenzyme, with its catalytic site in the extracellular space^61^ but recent evidence is making the case for a type III orientation in which the active site of an enzymatically active CD38 is facing the cytoplasm^62,63^. Hence, in light of these findings, ADPR production by CD38 does not seem to be limited to the extracellular space. Interestingly, a new TRPM2 superagonist, namely 2′-deoxy-ADPR, was recently identified in Jurkat T cells as an intracellular product of type III CD38 and found to be more potent than ADPR at inducing TRPM2-mediated whole-cell currents^64^. Moreover, intracellular production of ADPR by CD38 was reported in NK cells and shown to modulate Ca^2+^ signalling by gating TRPM2 channels and contributing to the anti-tumor activity of NK cells^65^. Hence, we propose that in the presence of CD38, the abnormally high concentration of intracelular NAD resulting from chronic inflammation is converted into ADPR, which induces enhanced and persistent TRPM2-mediated Ca^2+^ influx that may tilt the balance towards cell death. In *Cd38*^−/−^ mice, the increased concentration of intracellular NAD would stimulate the deacetylase activity of SIRT1 (and other sirtuins) while the low production of ADPR would attenuate TRMP2 channel activity. Both effects are likely to have an impact on the inflammatory and autoimmune processes triggered by pristane. Hence, pharmacological inhibition of CD38 activity or treatment with anti-CD38 antibodies could prevent some of the pristane-induced effects in WT mice. Interestingly, this mechanism does not seem to be operative in neutrophils, since no differences in frequencies and numbers of early-apoptotic pristane-elicited Ly6G^+^ neutrophils were detected in *Cd38*^−/−^ and *Trpm2*^*−/−*^ mice at 1- and 2-weeks post pristane treatment. One possible explanation for these results may be the more potent pro-inflammatory peritoneal environment in WT mice. Increased TNF-α production, which is significantly more pronounced in neutrophils, may stimulate ERK activation and decrease MCL-1 degradation in these cells and not in monocytes. Indeed, this is a well-established neutrophilic mechanism of protection from apoptosis^44^.

Apoptosis does not normally activate the immune system since apoptotic cells are rapidly cleared by phagocytes without the release of nucleosomes and with minimal inflammation^66^. However, inefficient clearance of dying cells can result in the accumulation of apoptotic cell remnants and lead to a process referred to as secondary necrosis^67^. Secondary necrosis can result in a permanent presence of cellular debris that can initiate pristane-induced lupus^2,41^ and systemic autoimmunity in SLE^67^. The pro-apoptotic effect of CD38 and TRPM2 expression in Ly6C^hi^ monocytes is particularly harmful in the pristane-induced model of lupus in which PECs lack highly phagocytic resident peritoneal macrophages (Tim4^+^ macs), and have low numbers of anti-inflammatory elicited macrophages (Tim4^−^CD138^+^Marco^+^ macs), which have increased phagocytic capacity for apoptotic cell clearance compared to Tim4^+^ macs^68^. Macrophages are the primary cell type responsible for the resolution of inflammation and the clearance of apoptotic debris in most tissues. Phagocytosis of apoptotic neutrophils by macrophages promotes a switch from a pro- to an anti-inflammatory macrophage phenotype^69^, which is clearly delayed or absent in pristane-induced lupus and other animal models of lupus^70^. In *Cd38*^−/−^ and *Trpm2*^−/−^ mice, the decreased pristane-induced apoptosis of Ly6C^hi^ monocytes may allow for a more effective clearance of apoptotic bodies and potentially harmful material compared to WT mice.

Interestingly, apoptotic cell engulfment activates nuclear receptors, such as Liver X receptors (LXR), which in turn induce the expression of the receptor tyrosine kinase Mer that is critical for phagocytosis^71^. In macrophages, CD38 expression is required for the control of bacterial infection mediated by LXR through a mechanism that involves consumption of NAD^72^. Moreover, agonist activation of LXR induces the transcriptional activation of CD38 synergistically with pro-inflammatory cytokines/mediators, such as TNF-α, IFN-γ, and lipopolysaccharide (LPS)^72^. Hence, it is possible that CD38 is required for other LXR-mediated activities beyond the control of infection.

Decreased clearance of apoptotic cells is a mechanism prevalent in lupus-prone mice^70^. While activation of LXR improves the clearance of apoptotic cells and ameliorates progression in lupus-prone B6^*lpr/lpr*^ mice, the lack of LXR leads to age-dependent systemic autoimmune disease^71^. Importantly, CD38 deficiency contributes to the exacerbation of the lupus-like disease in B6^*lpr/lpr*^ mice^73^, with clinical and immunological abnormalities resembling those in LXR-deficient mice^71^. Although the underlying mechanism has not yet been fully investigated, we speculate that the lack of CD38 in B6^*lpr/lpr*^ mice may affect the activation of LXR resulting in decreased clearance of apoptotic cells. We further speculate that in the peritoneum of pristane-treated WT mice, CD38-mediated LXR activation may be impaired due to the low number of peritoneal macrophages present. Therefore, WT mice would have to cope with a massive rate of apoptosis accompanied by defective clearance of apoptotic remnants. In *Cd38*^−/−^ mice, on the other hand, the lower number of apoptotic Ly6C^hi^ monocytes may allow for more effective clearance of apoptotic bodies and potentially harmful material despite a malfunctioning CD38-LXR axis^72^. Therefore, the pristane-elicited unbalanced cell environment in the peritoneum is crucial to the outcome of the lupus disease.

NAD-induced cell death (NICD), which acts through the ART2-P2X7 pathway^74,75^ is regulated by CD38. Indeed, lack of CD38 in ART2^+^ T cells results in increased NICD, which correlates with a significant reduction in Tregs and iNKT cells, even under steady-state conditions^76^. Moreover, CD38 deficiency accelerates diabetes type I (T1D) in NOD mice via a drastic reduction in regulatory CD4^+^ iNKT cells, which greatly affects the iNKT-mediated induction of tolerogenic dendritic cells (DCs) in specific niches, such as pancreatic lymph nodes^77^. Indeed, the loss of peripheral CD4^+^ iNKT cells in *Cd38*^−/−^ mice is due to ART2-dependent NICD^77^. In mice with a B6 genetic background, on the other hand, activated iNKT cells can boost a Th1 response by inducing the differentiation of DCs to an immunogenic or pro-inflammatory phenotype that support, rather than inhibit, AI4 T-cell-induced T1D^78^. Therefore, iNKT cells can play both suppressive and pathogenic roles in the regulation of T1D, in a genetically-determined manner. We have reported a similar mechanism in the pathogenesis of CIA, and showed that lack of CD38 in a B6 genetic background ameliorated the disease due to decreased iNKT cells in secondary lymphoid organs that were unable to boost a Th1 response^21^.

The concentration of extracellular NAD in the peritoneum may increase significantly in pristane-treated mice as a consequence of the high number of apoptotic cells. NAD may diffuse and reach local draining lymph nodes, or the tertiary lymphoid tissues, known as lipogranulomes, that are formed in response to pristane^79^. Hence, extracellular NAD may induce measurable ART2-dependent effects on the phenotype or function of T cells in these compartments^76^, a hypothesis that was not tested in this study. The mechanisms of apoptosis that induce a defective number of Ly6C^hi^ monocytes in *Cd38*^−/−^ PECs upon pristane treatment seem to be CD38-dependent and ART2-independent. Moreover, deficiency of CD38, but not of ART2, alters the pristane-elicited expression of ISGs. Regarding glomerular cellularity, 12-weeks pristane-treated *Art2*^−/−^ mice showed an increased response similar to their WT counterparts, while *Cd38*^−/−^*Art2*^−/−^ double knockout mice exhibited a decreased response similar to *Cd38*^−/−^ mice (data not shown). Therefore, ART2 deficiency does not seem to affect the development of pristane-induced lupus.

In summary, CD38 enhances the development of pristane-induced lupus by regulating the cell death of Ly6C^hi^ monocytes and Ly6C^lo^ monocytes/macrophages in a TRPM2-dependent and ART2-independent manner that worsens the early inflammatory response in the peritoneum. Our results highlight the importance of the immunoregulatory role of CD38 in inflammation and in the development of systemic autoimmunity leading to lupus, which should be considered when designing CD38-specific therapies for the treatment of inflammatory and autoimmune diseases.

## Methods

### Mice and pristane treatment

*Cd38*^−/−^, *Art2*^−/−^, and *Cd38*^−/−^*Art2*^−/−^ double knockout mice were backcrossed for 12 generations to the C57BL/6 J (B6) background^80–82^, bred and maintained under specific pathogen-free conditions at the IPBLN-CSIC Animal Facility in Granada, Spain. ARTC2 is a toxin-related, GPI-anchored ADP-ribosyltransferase expressed by murine T cells, also known as ART2^81,83^. Please note that ART2 is encoded by two closely related tandem genes in the mouse designated *Art2.1* and *Art2.2*. The ART2.1 and ART2.2 gene products are co-expressed by T cells and show similar enzymatic activities. The *Art2*^*−/−*^ mice are deficient for *Art2.1* and *Art2.2*^81^. For the sake of brevity “ART2” is used throughout this paper instead of “ART2.1 and ART2.2”. Wild-type C57BL/6 J mice were purchased from Charles River (Barcelona, Spain). *Trpm2*^−/−^ mice were kindly provided by Dr. Yasuo Mori from Kyoto University, Japan, and the generation of this mouse strain has been described^84^. Experimental mice received a single dose of 0.5 ml pristane (2,6,10,14-tetramethylpentadecane^1^; Sigma-Aldrich) that was filtered through a 0.25-μm filter and administered intraperitoneally (i.p.). Control mice received either a single dose of 0.5 ml saline or were left untreated. All experimental procedures involving animals at IPBLN-CSIC were approved by the Institutional Animal Care and Use Committee, which follows the ARRIVE guidelines^85^ in accordance with the U.K. Animals (Scientific Procedures, Act, 1986) and associated guidelines (EU Directive 2010/63/EU for animal experiments), and with the National Institutes of Health guide for the care and use of Laboratory animals (NIH Publications No. 8023, revised 1978). All the experiments with WT, *Cd38*^−/−^ and *Trpm2*^−/−^ mice described in Figs 3, S1 and S2 were carried out at the University of Alabama at Birmingham in the laboratory of Dr. Frances E. Lund, approved by the Institutional Animal Care and Use Committee, and performed according to guidelines outlined by the National Research Council.

### Flow cytometry and cell sorting

Flow cytometry analyses at IPBLN-CSIC were performed as previously described^86^. Before surface staining, peritoneal or peripheral blood cells were incubated with anti-mouse CD16/32 (Fc Block; BD Biosciences) for 10 min. Cells then were stained with an optimized amount of primary Ab or the appropriate isotype control for 10 min at room temperature before washing and re-suspending in staining media (PBS supplemented with 0.1% BSA and 2 mM EDTA). Ten thousand to 50,000 events per sample were acquired using a FACSCalibur flow cytometer (4-color, BD Biosciences) and analysed with FlowJo software (Tree Star, Inc.). The following conjugated mAbs were used: B220-FITC, CXCR4-APC, and CD11b-FITC (BioLegend), CD11b-APC, MHC-PE, CD11c-FITC, and CD8α-APC (Miltenyi Biotec), Gr1-PE, CD5-PerCP, Ly6C-PE, CD4-PerCP, Ly6C-FITC, and Ly6G-PE, Ly6G-PerCP (BD Biosciences). Unconjugated anti-mouse CCR2 mAb (clone MC-21) was used to detect surface expression of CCR2^87^. Briefly, cells were incubated with 5 μg/ml anti-mouse CCR2 mAb for 1 hour on ice, followed by 5 μg/ml biotinylated mouse anti-rat IgG2b (Clone RG7/11.1; BD Biosciences) for 30 min on ice. After blocking with 10% rat serum for 10 minutes, streptavidin-PE and a combination of directly labelled antibodies were added (CD11b-APC, Ly6C-FITC, and Ly6G-PerCP).

Cell sorting was performed using a FACSAria III flow cytometer (BD Biosciences). PECs from pools of 5 mice/group (2-weeks pristane-treated mice) were stained with CD11b-APC, Ly6C-FITC and Ly6G-PE mAbs. Doublets (FSC-H vs. FSC-A) were excluded and CD11b^+^ cells were gated in the singlet population prior to sorting. Ly6C^hi^ monocytes (CD11b^+^Ly6G^−^Ly6C^hi^), Ly6C^lo^ monocytes/macrophages (CD11b^+^Ly6G^−^Ly6C^lo^), and granulocytes (CD11b^+^Ly6G^+^Ly6C^int^) were sorted to 92–99.8% purity for cell culture or RNA isolation.

Flow cytometry analyses at UAB were performed using a FACSCanto II (BD Biosciences) flow cytometer. Peritoneal cells were incubated in staining media for 20 min on ice with a surface staining mix consisting of Ly6G-PE (clone 1A8; 1:200), CD19-APC (clone 1D3; 1:200), Ly6C-APC-Cy7 (clone AL-21; 1:200), CD11b-V500 (clone M1/70; 1:200) and Fc Block (1:1000) from BD Biosciences and CD3-Pacific Blue (Biolegend clone 17A2; 1:200) from Biolegend. Cells were then washed with ice-cold PBS and stained for apoptotic/necrotic cells using the FITC Annexin V Apoptosis Detection kit I (BD Biosciences). Briefly, cells were incubated with Annexin V-FITC (1:50) and 7-AAD (1:1000) in 1 × Annexin V binding buffer on ice for 15 min. The stained PECs were then diluted 1:2 with 1 × Annexin V binding buffer prior to acquisition on the flow cytometer. Single-color controls were generated by staining murine splenocytes with the following single mAbs in staining media on ice for 20 min: CD4-FITC (clone GK1.5; 1:200), CD4-PE (clone RM4-5; 1:200), CD8a-PerCP-Cy5.5 (clone 53-6.7; 1:200), CD4-PE-Cy7 (clone RM4-5; 1:200), CD45R/B220-APC-Cy7 (clone RA3-6B2; 1:200), and CD4-V500 (clone RM-4-5; 1:200) from BD Biosciences, and CD4-Pacific Blue (clone GK1.5; 1:200) and CD4-APC (clone RM4-5; 1:200) from BioLegend.

### Intracellular cytokine staining

Intracellular TNF-α was detected after culturing pristane-elicited PECs *in vitro* for 1 hour at a concentration of 2.5 × 10^6^ cells/ml in RPMI 1640 medium containing 10% fetal bovine serum (FBS), 20 ng/ml PMA and 1 μg/ml ionomycin, and stimulating them for 4 hours with 1 μg/ml Brefeldin A (BFA; Sigma-Aldrich) at 37 °C and 5% CO_2_. After stimulation, cells were surface stained, fixed and permeabilised using the Inside Stain Kit (Miltenyi Biotec), and stained with either TNF-α-FITC (Miltenyi Biotec) or isotype controls according to the manufacturer’s protocol for cells in suspension. Cells treated with PMA and ionomycin in the absence of BFA served as the negative controls. Cells were then washed twice in Perm/Wash buffer, resuspended in staining media, and analysed by flow cytometry.

### Cytokine ELISAs

The Bio-Plex Precision Pro Mouse Cytokine 3-Plex kit assay (Bio-Rad Laboratories) was used to simultaneously test cytokines IL-6, IL-12(p70) and TNF-α, and chemokine CCL2 (MCP-1). All 14 mouse IFN-α subtypes were measured using the Verikine Mouse Interferon-alpha ELISA (PBL Assay Science). Assays were performed according to the manufacturers’ protocols. Analyses of experimental data were carried out using five-parameter logistic curve fitting to standard analyte values.

### Chemotaxis assays

BM cells from WT and *Cd38*^−/−^ naïve mice were first stained with biotinylated CD115 antibody and MACS streptavidin microbeads (Miltenyi Biotec) and then positively selected on a MACS midi column. Purified monocytes were washed and re-suspended in HBSS (Ca^2+^, Mg^2+^) supplemented with 1% FBS. Chemotaxis assays were performed in 24-well Transwell plates (Costar, Cambridge, MA) with 5-μm pore size polycarbonate filters as previously described^7^. Briefly, CCL2 or CXCL12 chemokines were diluted in HBSS, and placed in the lower chamber of the Transwell while cells were added to the upper chamber. For mouse monocyte assays, 5 × 10^5^ cells/Transwell were incubated at 37 °C for 2 hours. The transmigrated cells were collected from the lower chamber, fixed, and counted on a flow cytometer.

### Western-blotting experiments

1% NP-40 lysates from 2-, 4- and 8-weeks pristane-elicited PECs were run on Criterion TGX stain-free gels (Bio-Rad Laboratories), and immediately after electrophoretic separation was complete, the gels were activated with UV-light using the Gel Doc EZ system (Bio-Rad Laboratories) and the proteins were transferred to PVDF membranes using the Trans-Blot Turbo system (Bio-Rad Laboratories). Once the blotting step was complete, a stain-free blot image of the PVDF membrane was acquired prior to blocking with 5% non-fat dry milk in tris (hydroxymethyl) aminomethane-buffered saline containing 0.1% polysorbate surfactant (TBST) for 1 hour at room temperature. The membranes were then incubated overnight at 4 °C with either a polyclonal rabbit antibody specific for the 17 kDa subunit of active caspase-3 (ImmunoStep, Salamanca, Spain), monoclonal antibodies specific for phospho-ERK1/2 (Thr^185^/Tyr^187^), total Erk1/2, phospho-AKT (Ser^473^) and total AKT (Cell Signaling Technology, Massachusetts), or a polyclonal antibody specific for MCL-1 (Santa Cruz Biotechnology). The membranes were washed several times with TBST and incubated with horseradish peroxidase-conjugated goat anti-rabbit or anti-mouse secondary antibodies (Cell Signaling Technology) accordingly, for 1 hour. The blots were washed again and developed by chemiluminescence with substrate Immun-Star HRP substrate (Bio-Rad Laboratories) using a Chemidoc image analyzer (Bio-Rad Laboratories). The Stain-Free technology was used as a loading control method^88^.

### Polarization of BM-derived macrophages

BM cells from *Cd38*^−/−^ or WT naïve mice were differentiated towards anti-inflammatory macrophages by culturing them for 7 days in the presence of murine M-CSF as previously described^32^. Subsequent analyses of the mouse M-Mϕ-specific gene markers *Emr1*, *Cd163*, *Cnrp1*, and *Ctla2*, and the GM-Mϕ-specific gene markers *Nos2*, *Ccr7*, and *Cd11c* were performed as previously described^32^.

### Serological studies

Serum levels of IgG anti-ssDNA autoantibodies were measured by ELISA, and the results were expressed in titration units (U/ml) extrapolated from a standard curve generated with a serial dilution of a serum pool from 6- to 8-month-old MRL-*Fas*^*lpr*^ mice as previously described^89^. Total IgG serum levels were measured by ELISA as previously described^89,90^. The mouse anti-nRNP total Ig ELISA kit (Alpha Diagnostic International) was used to quantify total nRNP-specific antibody (IgG, IgA, and IgM) in sera following the manufacturer’s instructions. To quantify positive antibody levels, a Positive index was first determined by calculating the net OD mean + 3 SD of the Control/Non-immune samples. Each sample net OD was then divided by the Positive Index. A value above 1.0 was considered a Positive Antibody Activity, while a value below 1.0 was considered a Negative Antibody Activity.

### Assessment of glomerulonephritis

Kidneys were excised from experimental and control mice, fixed in 4% paraformaldehyde, and embedded in paraffin. Glomerular cellularity was evaluated by counting the number of nuclei per glomerular cross-section (*n* = 5 mice/group or as indicated, 8 glomeruli/mouse) after staining with hematoxylin and eosin (H&E)^37^. Nuclei number analysis was automated using the open-source ImageJ platform Fiji^91^.

### Real-time quantitative PCR

Quantitative PCR (Q-PCR) was performed as previously described^86^. In brief, total RNA was extracted from 10^6^ peritoneal cells using TRIzol reagent (Invitrogen), and cDNA was synthesized using the Superscript II First-Strand Synthesis kit (Invitrogen) following the manufacturer’s protocol. SYBR Green Q-PCR analysis was performed using an Opticon II thermocycler (Bio-Rad Laboratories). Amplification conditions were as follows: 95 °C for 10 min, followed by 45 cycles of 94 °C for 15 s, 60 °C for 25 s, and 72 °C for 25 s. After the final extension (72 °C for 10 min), a melting-curve analysis was performed to ensure specificity of the products. Primer sequences for *Ccl2*, *Ccl7*, *Ccl12*, *Ccr2*, *Cxcl12*, *Cxcr4*, *Isg15*, *Irf7*, *Ly6G*, *Mx1*, *Tlr7*, *Tlr9*, and *Tnfα* are listed in Supplemental Table S1.

### Q-PCR data analysis

Cycle threshold (Ct) values were processed and normalized with R 3.2.2 (R-Core 2015) using the comparative Ct method^92^ based on the comparison of the Ct values of the gene of interest normalized to the Ct values of the housekeeping internal control genes. Following normalization, a quality step was performed to evaluate the replicate’s performance. Principal component analysis (PCA), heat-maps, cluster dendrograms and boxplots of raw and normalized Ct values were assessed using the HTqPCR platform (Bioconductor)^93^.

### Statistical analysis

To measure differential expression, limma package^94^ was used for statistical analysis and false discovery rate (FDR) correction^95^. Fold change was computed based on the 2^−ΔΔCt^ formula^96^ shown below:

$${2}^{-{\rm{\Delta }}{\rm{\Delta }}\mathrm{Ct}}=[({\rm{Ct}}\,{\rm{gene}}\,{\rm{of}}\,{\rm{interest}}\mbox{--}{\rm{Ct}}\,{\rm{internal}}\,{\rm{control}}){\rm{sample}}\,{\rm{A}}\,-\,[({\rm{Ct}}\,{\rm{gene}}\,{\rm{of}}\,{\rm{interest}}\mbox{--}{\rm{Ct}}\,{\rm{internal}}\,{\rm{control}})\,\,\,\,\times \,{\rm{sample}}\,{\rm{B}}]$$

Genes having a FDR lower than 0.05 were designated as differentially expressed. For other phenotypical and functional assays, statistical analyses were performed using the Prism 7 software (GraphPad).

## Electronic supplementary material

Supplementary information
