# Supplementary material for: CD38 promotes pristane-induced chronic inflammation and increases susceptibility to experimental lupus by an apoptosis-driven and TRPM2-dependent mechanism
Source: Sci Rep. 2018 Feb 20;8:3357. doi: 10.1038/s41598-018-21337-6 (PMC5820326; doi:10.1038/s41598-018-21337-6)
Supplement: Supplementary file 1 — Supplementary information [file 41598_2018_21337_MOESM1_ESM.pdf]

CD38 promotes pristane-induced chronic inflammation and increases susceptibility to experimental lupus by an apoptosis-driven and TRPM2-mediated mechanism

Sonia García-Rodríguez, Antonio Rosal-Vela, Davide Botta, Luz M. Cumba Garcia, Esther Zumaquero, Verónica Prados-Maniviesa, Daniela Cerezo-Wallis, Nicola Lo Buono, José-Ángel Robles Guirado, Salvador Guerrero, Elena González-Paredes, Eduardo Andrés-León, Ángel Corbí, Matthias Mack, Friedrich Koch-Nolte, Ramón Merino, Mercedes Zubiaur, Frances E. Lund, and Jaime Sancho.

Supplementary information

Results

Expression of Type I IFN-inducible genes in response to pristane treatment is CD38-dependent and ART2-independent. It has previously been reported that within 2 weeks of pristane treatment there is an increased expression of ISGs that is concurrent with the accumulation of IFN-I-producing CD11b^{+}Ly6C^{hi} monocytes in the PC [1]. Indeed, pristane elicits IFN-I production, monocyte recruitment and autoantibody production, which have been shown to be dependent on the Toll-like receptor (TLR) 7 pathway [2]. Indeed, the pro-inflammatory pristane-elicited Ly6C^{hi} monocytes that infiltrate the peritoneum express high levels of TLR7 and CCR2 [2]. In addition, TLR9 has also been suggested to play a role in the mouse model of pristane-induced lupus, although its role seems to be more complex than that of TLR7 [2-5]. In our initial approach, we measured the expression of ISGs in PECs from 2-weeks pristane-treated WT, *Cd38*$^{-/-}$, *Art2*$^{-/-}$, and *Cd38*$^{-/-}$*Art2*$^{-/-}$ mice and compared it to those in PECs from their control saline-treated counterparts. Pristane-elicited PECs had increased expression of ISGs, most notably *Ccl12* (MCP-5), *Ccl2* (MCP-1), *Ccl7* (MCP-3), *Irf7*, *Isg15*, and *Mx1*

(Fig. S4). *Ccr2* expression was also significantly increased in all 4 groups of pristane-treated mice, while increased *Tlr7* expression was only observed in WT mice, and decreased *Tlr9* expression was only observed in *Cd38*$^{-/-}$ mice. Importantly, PECs from 2-weeks pristane-treated *Cd38*$^{-/-}$ mice had decreased expression of all measured ISGs and *Tlr9* compared to pristane-elicited WT PECs (Fig. S4e). We also observed similar statistically-significant differences in gene expression levels between pristane-elicited *Cd38*$^{-/-}$ and *Art2*$^{-/-}$ PECs (Fig. S4g). The defective SIG expression signature in *Cd38*$^{-/-}$ PECs correlated with that reported in pristane-elicited *Tlr7*$^{-/-}$, *Ifnar*$^{-/-}$ and *Irf5*$^{-/-}$ PECs [2,6,7], and strongly suggests that CD38 deficiency, but not ART2 deficiency, alters IFN-stimulated ISG expression.

Distinct gene expression profile in sorted pristane-elicited Ly6C^{hi} monocytes, Ly6C^{lo} monocytes/macrophages and Ly6G^{+} neutrophils from WT and *Cd38*$^{-/-}$ mice.

In agreement with previously published studies [2], pristane-elicited Ly6C^{hi} monocytes expressed higher levels of *Tlr7* than any other peritoneal cell subset analysed, including Ly6C^{lo} monocytes (Fig. S8a). However, no difference was observed in *Tlr7* gene expression between WT and *Cd38*$^{-/-}$ mice. Likewise, Ly6C^{hi} monocytes displayed the highest *Tlr9* expression out of the 3 PEC populations analysed, with increased expression in *Cd38*$^{-/-}$ mice (Fig. S8b). In contrast, *Gr1* expression was almost exclusively detected in Ly6G^{+} neutrophils as expected (Fig. S8c). Moreover, expression of *Tnf-*α was higher in neutrophils than in the other cell types and showed an increased expression in WT mice (Fig. S8d). With regards to ISGs, the expression of *Isg15* and *Irf7* were detected primarily in Ly6G^{+} neutrophils, while *Mx1* and *Ccl2* were detected in Ly6C^{hi} monocytes with a distinct cell profile (Fig. S8e-h). Thus, expression of *Isg15,* and to a lesser extent *Irf7,* was increased in cells from pristane-treated WT mice, while *Mx1,* and *Ccl2* expression was increased in cells from pristane-treated *Cd38*$^{-/-}$ mice. In

contrast, Ly6C^{lo} monocytes showed a relatively low expression of all these genes. Overall, these results suggest that the observed defective secretion of TNF-α by PECs isolated from 2-weeks pristane-treated *Cd38*$^{-/-}$ mice in response to TLR7 agonist R848 was not due to a defective transcription of the *Tlr7* gene, and defective secretion of CCL2 did not reflect diminished transcription of the *Ccl2* gene by *Cd38*$^{-/-}$ Ly6C^{hi} monocytes upon pristane treatment.

***In vitro* chemotaxis of BM Ly6C^{hi} monocytes to CCL2 and CXCL12 is CD38-independent.** Previous studies have shown that chemotaxis of human monocytes to the FPR ligand fMLP was independent of cADPR, while chemotaxis to the CCR1/CCR5 ligands RANTES and MIP-1, and to the CXCR4 ligand SDF-1, was regulated by cADPR [8]. Unlike the extensive chemotactic studies performed with human monocytes, those with murine monocytes are limited primarily due to the difficulty in obtaining the large numbers of highly purified monocytes needed for such analyses. Altered recruitment of inflammatory Ly6C^{hi} monocytes to sites of *Listeria* infection in *Cd38*$^{-/-}$ mice could be explained in part by an impaired migration of these cells [9], so we assessed the chemotaxis of mouse monocytes to CCL2 and CXCL12.

We purified Ly6C^{hi} monocytes from the bone marrow of WT and *Cd38*$^{-/-}$ mice by positive selection using anti-CD115 antibody. Purity was assessed by flow cytometry and found to be ≥70% (Fig. S9a). The responses of WT and *Cd38*$^{-/-}$ monocytes to chemoattractants CCL2 and CXCL12 were measured *in vitro*, and found to be similar between the two groups (Fig S9b). Therefore, it is unlikely that the reduced accumulation of Ly6C^{hi} monocytes in the peritoneum of pristane-treated *Cd38*$^{-/-}$ mice was due to an intrinsically defective chemotactic response to these chemokines.

***In vivo* migration of Ly6G$^+$ neutrophils to the peritoneum in response to zymosan challenge is CD38-independent.** WT and *Cd38*$^{-/-}$ mice were injected i.p. with 0.5 mg of zymosan A following the model of acute self-limited inflammation [10], and the *in vivo* migratory capabilities of Ly6G$^+$ neutrophils were assessed 4-hours post treatment. In agreement with previously published data [10], PECs consisted predominantly of Ly6G$^+$ neutrophils during the initiation phase (Fig. S9c). However, no major differences in frequencies of Ly6G$^+$ neutrophils were observed between *Cd38*$^{-/-}$ (83.8% ± 0.8) and WT (81.9% ± 2.5) mice (Fig. S9c and S9d). Zymosan-treated mice also had an increased frequency of peritoneal Ly6C^{hi} monocytes (5-10%) compared to untreated control mice (~1%). Moreover, resident macrophages, which in control mice made up >90% of the CD11b$^+$ cells (Fig. S9c, left panel), sharply decreased in number and frequency following zymosan treatment. We also measured the frequencies of Ly6G$^+$ neutrophils in the spleen and peripheral blood of zymosan-treated mice, and no difference was detected between *Cd38*$^{-/-}$ and WT mice (data not shown), suggesting a normal mobilization of neutrophils from the BM to the periphery in *Cd38*$^{-/-}$ mice. Hence, our data suggest that the trafficking of neutrophils in *Cd38*$^{-/-}$ mice is not impaired in the early phase of this model of acute inflammation.

**Target gene** **(SwissProt acc. no.)**	**Forward primer (5´-3´)**	**Reverse primer (5´-3´)**	**Ref./Qiagen Cat. No.**
Ccl7 (Q03366)	gatctctgccacgcttctgt	atagcctcctcgacccactt	[11]
Ccl12 (Q62401)	gtcctcaggtattggctgga	cactggctgcttgtgattct	[11]
Ccl2 (P10148)	aggtccctgtcatgcttctg	ggatcatcttgctggtgaat	[1]
Tlr7 (P58681)	gctgtgtggtttgtctggtg	cccctttatctttgctttcc	[12]
Tlr9 (Q9EQU3)	gaaagcatcacccacaccaa	acaagtccacaaagcgaagg	[12]
Isg15 (Q64339)			QT01772876
Irf7 (P70434)			QT00245266
Mx1 (P09922)			QT01064231
Ccr2 (P51683)			QT02522849
Tnf (P06804)			QT00104006
Ly6g (P35461)			QT00529655
Cxcr4 (P70658)			QT00249305
Cxcl12 (P40224)			QT00161112
Tbp (P29037)	cggtcgcgtcattttctc	gggttatcttcacacaccatga	Primer3 software: [13]

Supplementary Table S1. Primers used for gene amplification in the Q-PCR experiments

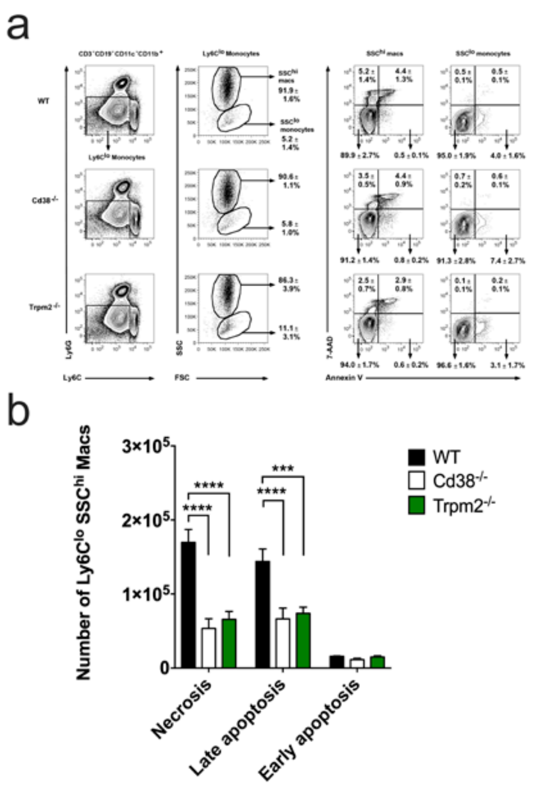

Figure S1

Supplementary Figure S1. Phenotypic characterization of pristane-elicited peritoneal Ly6C^{lo} cell subsets. (a) Representative flow cytometry plots showing gating and quadrant strategies for the identification of apoptotic/necrotic peritoneal Ly6C^{lo}SSC^{hi} (macrophages - macs) and Ly6C^{lo}SSC^{lo} (monocytes) cell subsets from WT, *Cd38$^{-/-}$* and *Trpm2$^{-/-}$* mice at 1-week post-pristane treatment. Frequencies of Ly6C^{lo}SSC^{hi} macs and Ly6C^{lo}SSC^{lo} monocytes are shown as percentages of the parent CD3^{-}CD19^{-}CD11c^{-}CD11b^{+}Ly6C^{lo} cells. Frequencies of live (AnnV^{-}7-AAD^{-}), early apoptotic (AnnV^{+}7-AAD^{-}), late apoptotic/dead (AnnV^{+}7-AAD^{+}) and necrotic (AnnV^{-}7-AAD^{+}) cells are shown as percentages of the Ly6C^{lo}SSC^{hi} and Ly6C^{lo}SSC^{lo} parent cell populations. **(b)** Numbers of early apoptotic, late apoptotic/dead and necrotic

Ly6C^{lo}SSC^{hi} cells from 1-week pristane-treated WT, *Cd38*$^{-/-}$ and *Trpm2*$^{-/-}$ mice. Data are shown as the mean ± SD (n = 5 mice/group). P values were determined by 2-way ANOVA with the Dunnett's multiple comparisons test. ****P*<0.001 *****P*<0.0001.

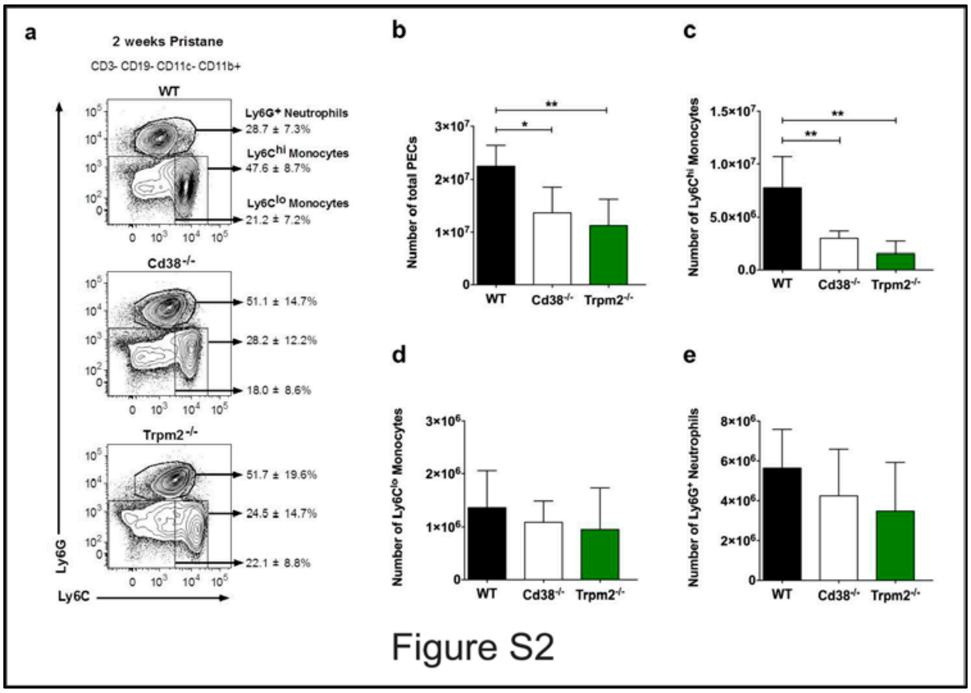

Supplementary Figure S2. Phenotypic similarities between pristane-treated *Cd38*$^{-/-}$ and *Trpm2*$^{-/-}$ mice. (a) Representative flow cytometry plots showing gating strategy for the identification of peritoneal Ly6C^{hi} monocytes, Ly6C^{lo} monocytes/macrophages and Ly6G^{+} neutrophils from WT, *Cd38*$^{-/-}$ and *Trpm2*$^{-/-}$ mice at 2-weeks post-pristane treatment. Frequencies are shown as percentages of the parent CD3^{-}CD19^{-}CD11c^{-} CD11b^{+} cells. **(b-e)** Numbers of total PECs **(b)**, Ly6C^{hi} monocytes **(c)**, Ly6C^{lo} monocytes/macrophages **(d)** and Ly6G^{+} neutrophils **(e)** from 2-weeks pristane-treated WT, *Cd38*$^{-/-}$ and *Trpm2*$^{-/-}$ mice. Data are shown as the mean ± SD (n = 7 mice/group). P values were determined using a two-tailed Student’s t-test. **P*<0.05 ***P*<0.01.

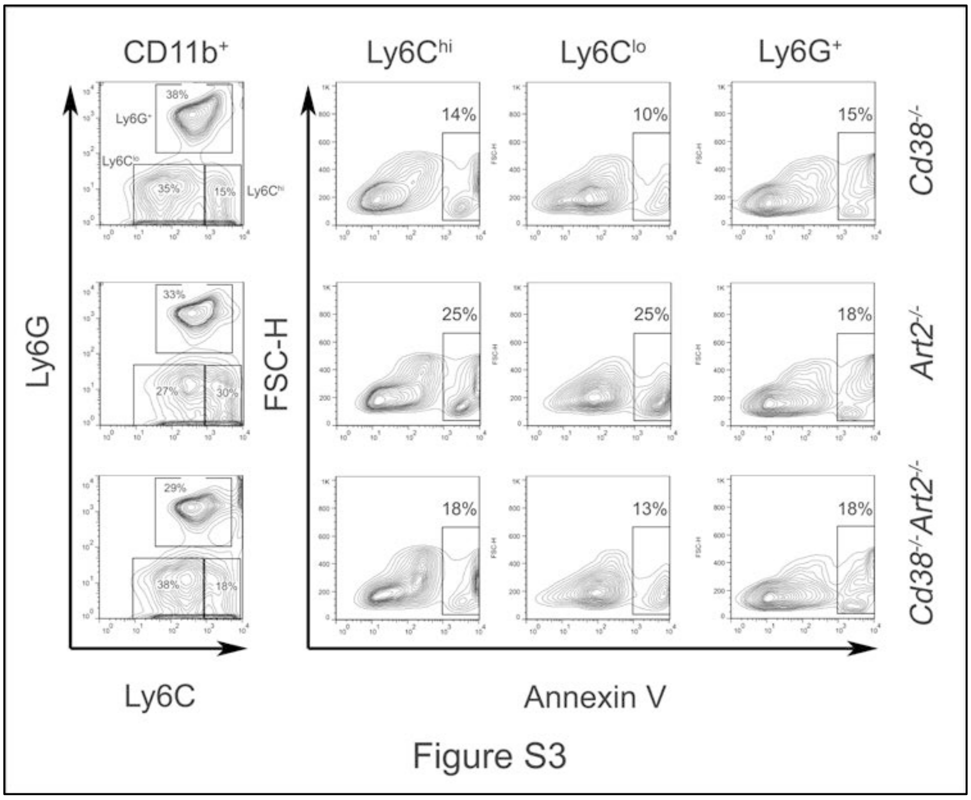

Supplementary Figure S3. Phenotypic differences between pristane-treated *Cd38*$^{-/-}$ and *Art2*$^{-/-}$ mice. Representative flow cytometry plots showing gating strategy and detection of apoptotic (Annexin V^{+}) peritoneal Ly6C^{hi} monocytes, Ly6C^{lo} monocytes/macrophages and Ly6G^{+} neutrophils from *Cd38*$^{-/-}$, *Art2*$^{-/-}$ and *Cd38*$^{-/-}$*Art2*$^{-/-}$ mice at 2-weeks post-pristane treatment. Frequencies are shown as percentages of the parent CD11b^{+} cells. The flow cytometry analysis was carried out using pooled PECs (n = 5 mice/group).

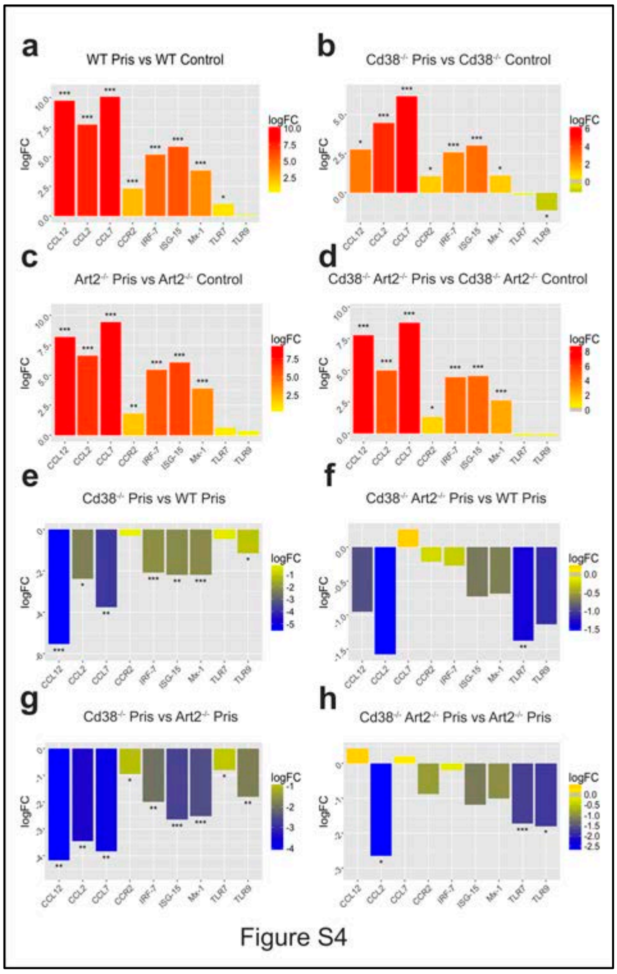

Supplementary Figure S4. Pristane-induced expression of Type I IFN-stimulated genes (ISGs) in PECs is CD38-dependent but ART2-independent. (a-h) Q-PCR analysis of *Ccl12*, *Ccl2*, *Ccl7*, *Ccr2*, *Irf7*, *Isg15*, *Mx1*, *Tlr9*, and *Tlr7* gene expression in PECs from 2-weeks pristane-treated vs. non-treated WT mice **(a)**, pristane-treated vs. non-treated *Cd38*$^{-/-}$ mice **(b)**, pristane-treated vs. non-treated *Art2*$^{-/-}$ mice **(c)**, pristane-treated vs. non-treated *Cd38*$^{-/-}$*Art2*$^{-/-}$ mice **(d)**, pristane-treated *Cd38*$^{-/-}$ mice vs. pristane-treated WT mice **(e)**, pristane-treated *Cd38*$^{-/-}$*Art2*$^{-/-}$ mice vs. pristane-treated WT mice **(f)**, pristane-treated *Cd38*$^{-/-}$ mice vs. pristane-treated *Art2*$^{-/-}$ mice **(g)**, and pristane-treated *Cd38*$^{-/-}$*Art2*$^{-/-}$ mice vs. *Art2*$^{-/-}$ mice **(h)**. Data are shown as logFC [= mean(log2(Group1)) - mean(log2(Group2)] (n = 4-6 mice/group). Asterisks represent the significance of the adjusted *P* values for multiple testing. ***P< 0.0001 **P< 0.001 *P<0.05.

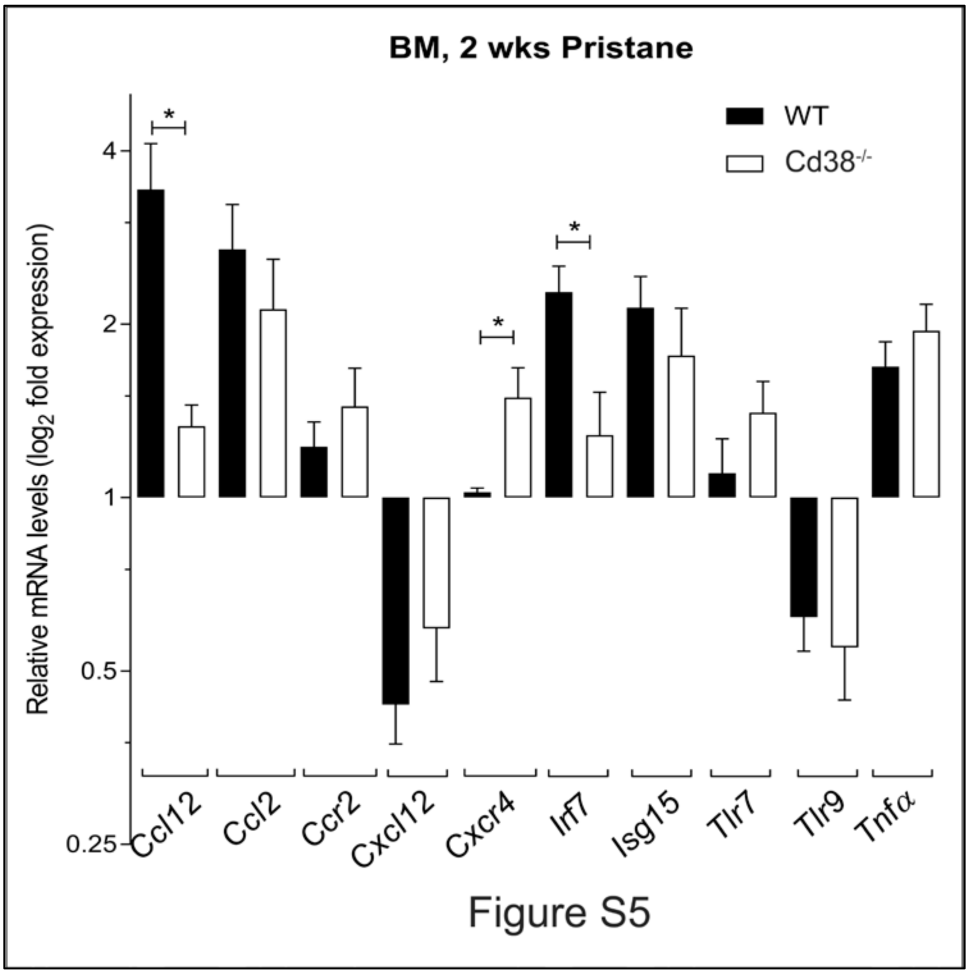

Supplementary Figure S5. Similar expression of ISGs and TNF-α-regulated genes in pristane-elicited BM cells from WT and *Cd38$^{-/-}$* mice. Q-PCR analysis of *Ccl12*, *Ccl2*, *Ccr2*, *Cxcl12*, Cxcr4, *Irf7*, *Isg15*, Tlr7, *Tlr9*, and *Tnf-α* gene expression in BM cells from 2-weeks pristane-treated vs. non-treated WT mice. Data represent the mean $\log_2$ of the fold increase relative to gene expression in non-treated WT mice ± SE (n = 6 mice/group). P values were determined using a two-tailed Student's t-test. **P*<0.05.

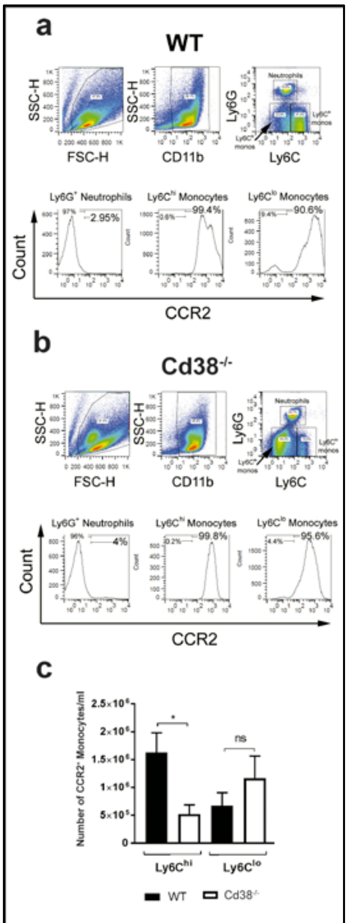

Figure S6

Supplementary Figure S6. **Decreased number of pristane-elicited CCR2^{+} Ly6C^{hi} monocytes in *Cd38$^{-/-}$* mice**. **(a, b)** Representative flow cytometry plots showing gating strategy and CCR2 expression level in peritoneal Ly6C^{hi} monocytes, Ly6C^{lo} monocytes/macrophages and Ly6G^{+} neutrophils from WT **(a)** and *Cd38$^{-/-}$* **(b)** mice at 2-weeks post-pristane treatment. Frequencies of CCR2^{+} cells are shown as percentages of each respective parent population. **(c)** Numbers of peritoneal CCR2^{+} Ly6C^{hi} monocytes and Ly6C^{lo} monocytes/macrophages from 2-weeks pristane-treated WT and *Cd38$^{-/-}$* mice. Data are shown as the mean ± SE (n = 4 mice/group) and are representative of 3 independent experiments. P values were determined using a two-tailed Student's t-test. **P*<0.05.

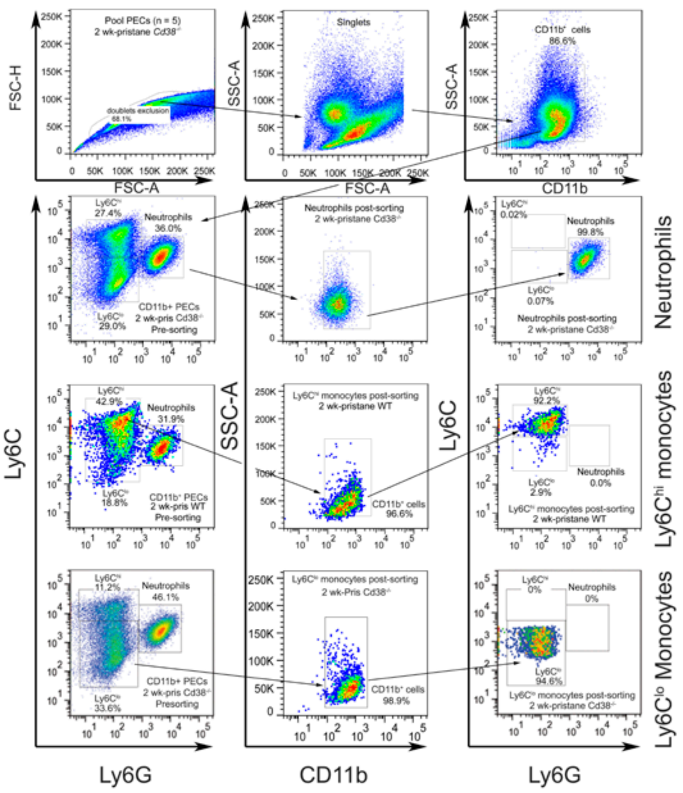

Figure S7

Supplementary Figure S7. Gating and sorting strategy to obtain pure pristane-elicited peritoneal Ly6C^{hi} monocytes, Ly6C^{lo} monocytes/macrophages and Ly6G^{+} neutrophils from WT and *Cd38*$^{-/-}$ mice. PECs were harvested and pooled from 2-weeks pristane-treated WT and *Cd38*$^{-/-}$ mice (5 mice/group), processed and stained for flow cytometry analyses as described in Methods. The cell sorting strategy involved the exclusion of doublet cells (FSC-H vs. FSC-A) and subsequent gating of CD11b^{+} cells (SSC-A vs. Cd11b) prior to the selection of Ly6C^{hi} monocytes, Ly6C^{lo} monocytes/macrophages and Ly6G^{+} neutrophils for cell sorting. The percent purity and SSC characteristic of each sorted cell population is indicated. In some panels, large-dot plots are shown to better show the high purity of the post-sort cell populations. Data are representative of 6 independent experiments. Sorted cells were used for gene expression

profiling (see Fig. S8) and for *in vitro* stimulation experiments/cytokine production (data not shown).

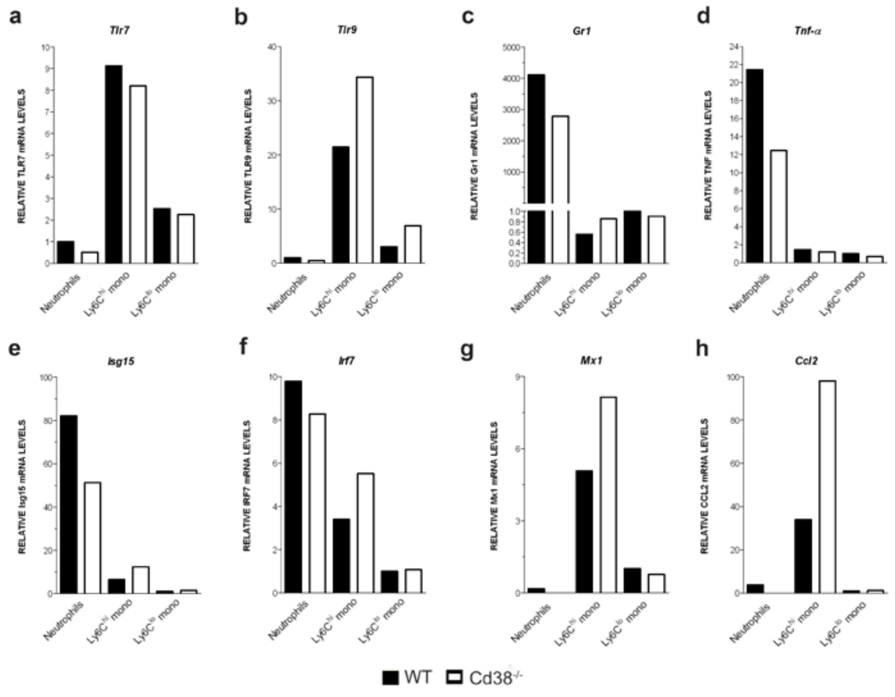

Figure S8

Supplementary Figure S8. **Distinct gene expression profiles between pristane-elicited peritoneal Ly6C^{hi} monocytes, Ly6C^{lo} monocytes/macrophages and Ly6G^{+} neutrophils from WT and *Cd38*$^{-/-}$ mice**. Q-PCR analysis of *Tlr7* **(a)**, *Tlr9* **(b)**, *Gr1* **(c)**, *Tnf-α* **(d)**, *Isg15* **(e)**, *Irf7* **(f)**, *Mx1* **(g)** and *Ccl2* **(h)** gene expression in peritoneal Ly6C^{hi} monocytes, Ly6C^{lo} monocytes/macrophages and Ly6G^{+} neutrophils sorted from pooled PECs harvested from WT (closed bars) and *Cd38*$^{-/-}$ (open bars) mice at 2-weeks post-pristane treatment (n = 5 mice/group). Data represent the fold increase relative to gene expression in WT Ly6G^{+} neutrophils (panels **a**, **b**, **g**, and **h**) or in WT Ly6C^{lo} monocytes

(panels **c**, **d**, **e**, and **f**). Data are representative of 3 independent experiments (2 experiments for WT vs. *Cd38*$^{-/-}$ and 1 experiment for *Art2*$^{-/-}$ vs. *Cd38*$^{-/-}$).

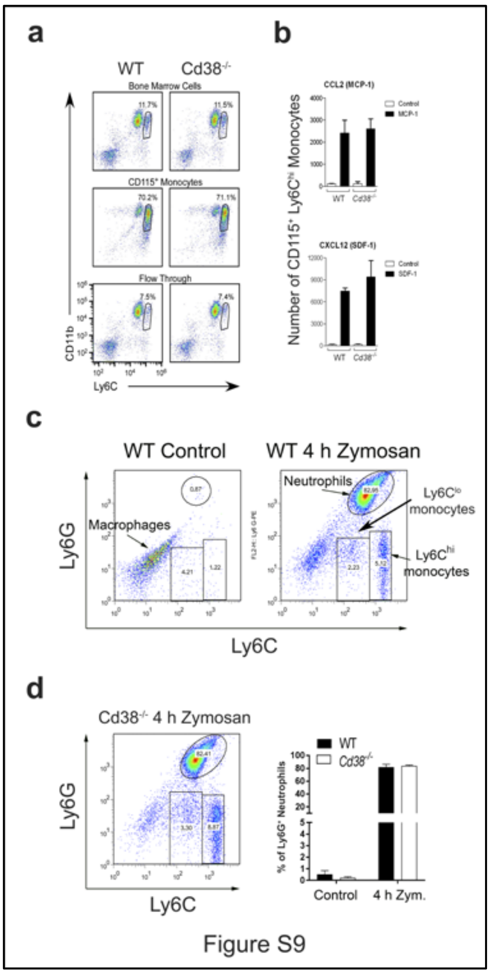

Supplementary Figure S9. Chemotaxis of BM Ly6C^{hi} monocytes to CCL2 and CXCL12, and zymosan-induced migration of BM Ly6G^{+} neutrophils to the peritoneum are CD38-independent. (a) Representative flow cytometry plots showing gating and frequencies of Cd11b^{+} Ly6C^{hi} monocytes in total BM cells (upper plots), CD115^{+} cells isolated by immunomagnetic positive selection (middle plots) and the flow-through (non-bound) BM cells (lower plots) from non-treated naïve WT and *Cd38*$^{-/-}$ mice. **(b)** Number of isolated WT and *Cd38*$^{-/-}$ BM CD115^{+} Ly6C^{hi} monocytes that chemotaxed to CCL2/MCP-1 and CXCL12/SDF-1 *in vitro* (closed bars) compared to non-stimulated cell controls (open bars). Data are representative of >4 independent

experiments and are shown as the mean ± SD (n = 3 wells/group). **(c, d)** Representative flow cytometry plots showing gating and frequencies of peritoneal Ly6C^{hi} monocytes, Ly6C^{lo} monocytes and Ly6G^{+} neutrophils from non-treated naïve WT mice (**c**, left plot), and from 4-hours zymosan-treated WT (**c**, right plot) and *Cd38*$^{-/-}$ (**d**, left plot) mice. Frequencies are shown as percentages of the parent CD11b^{+} cell populations. The distribution of resident peritoneal macrophages is indicated in the non-treated naïve WT plot. The frequencies of WT (closed bars) and *Cd38*$^{-/-}$ (open bars) peritoneal Ly6G^{+} neutrophils from non-treated naïve and zymosan-treated mice are graphed (**d**, right panel). Data are shown as the mean ± SD (n = 3 mice/group).

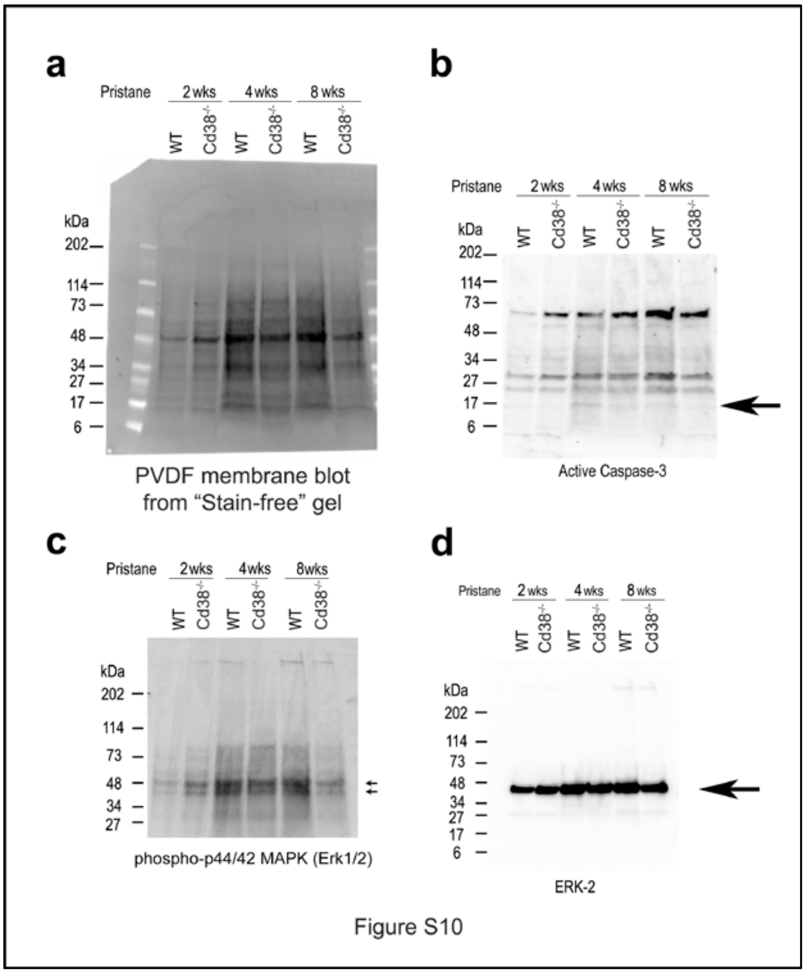

Supplementary Figure S10. Images of the non-cropped stain-free and Western blot membranes generated for the detection of active caspase-3 and phosphor-ERK1/2. **(a)** A stain-free blot image of the PVDF membrane acquired prior to Western blotting of PEC protein extracts from WT and *Cd38*$^{-/-}$ mice at 2-, 4- and 8-weeks post-pristane treatment, and which was used as the protein loading control. **(b)** Western blot analysis of the same membrane shown in panel (a) carried out using a polyclonal antibody specific for active caspase-3, a 17 kDa protein band indicated by an arrow. **(c)** Western blot analysis carried out using the E10 monoclonal antibody specific for phospho-ERK1/2 (Thr^{185}/Tyr^{187}). The corresponding 42 KDa and 44 KDa bands are indicated by arrows. **(d)** Western blot analysis carried out using an antibody specific for total ERK-2.

The corresponding band is indicated by an arrow. Each lane represents pooled protein extracts from 3 mice/group.

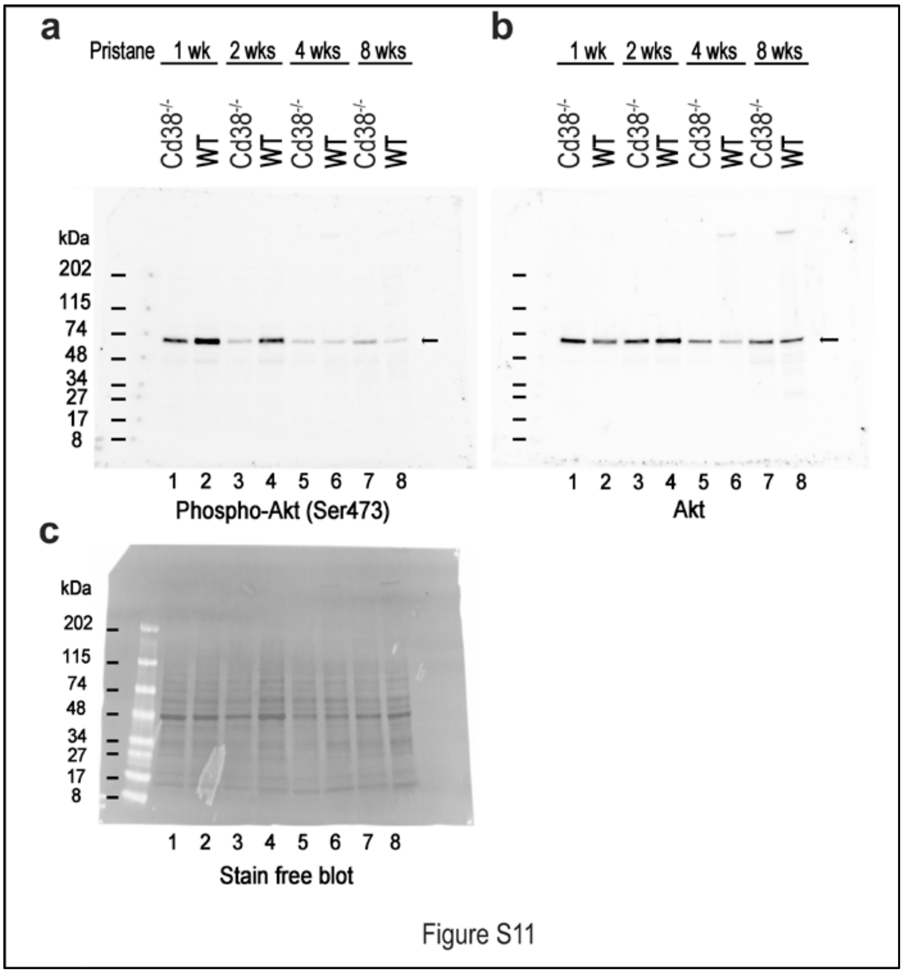

Supplementary Figure S11. Images of the non-cropped stain-free and Western blot membranes generated for the detection of total and phospho-AKT. (a, b) Western blot analysis of PEC protein extracts from WT and *Cd38*$^{-/-}$ mice at 2-, 4- and 8-weeks post-pristane treatment carried out using an antibody specific for Phospho-AKT (Ser^{473}) **(a)** and for total AKT **(b)**. **(c)** A stain-free blot image of the PVDF membrane acquired prior to Western blotting that was used as the protein loading control. Each lane represents pooled protein extracts from 3 mice/group.

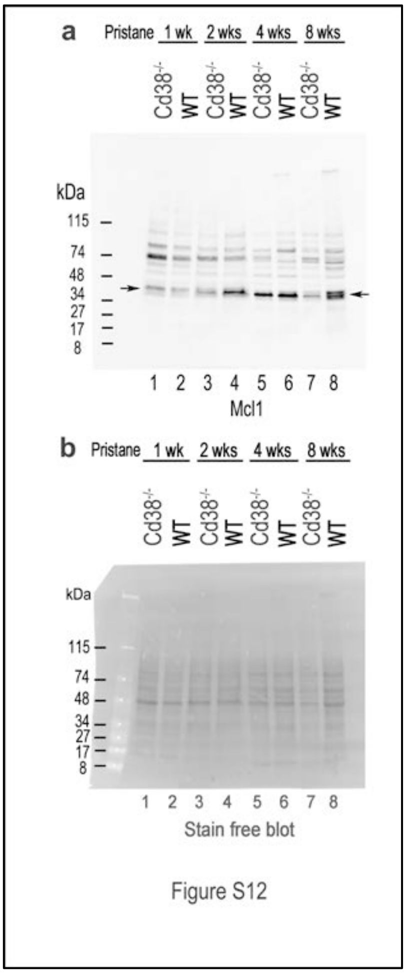

Supplementary Figure S12. Images of the non-cropped stain-free and Western blot membrane generated for the detection of MCL1. (a) Western blot analysis of PEC protein extracts from WT and *Cd38*$^{-/-}$ mice at 2-, 4- and 8-weeks post-pristane treatment carried out using a polyclonal antibody specific for MCL-1. **(d)** A stain-free blot image of the PVDF membrane acquired prior to Western blotting that was used as the protein loading control. Each lane represents pooled protein extracts from 3 mice/group.

References:

1 Lee, P. Y. *et al.* A novel type I IFN-producing cell subset in murine lupus. *Journal of immunology* **180**, 5101-5108 (2008).

2 Lee, P. Y. *et al.* TLR7-dependent and FcgammaR-independent production of type I interferon in experimental mouse lupus. *J Exp Med* **205**, 2995-3006, doi:10.1084/jem.20080462 (2008).

3 Thibault, D. L. *et al.* Type I interferon receptor controls B-cell expression of nucleic acid-sensing Toll-like receptors and autoantibody production in a murine model of lupus. *Arthritis research & therapy* **11**, R112, doi:10.1186/ar2771 (2009).

4 Summers, S. A. *et al.* TLR9 and TLR4 are required for the development of autoimmunity and lupus nephritis in pristane nephropathy. *Journal of autoimmunity* **35**, 291-298, doi:10.1016/j.jaut.2010.05.004 (2010).

5 Santiago-Raber, M. L., Baudino, L. & Izui, S. Emerging roles of TLR7 and TLR9 in murine SLE. *Journal of autoimmunity* **33**, 231-238, doi:S0896-8411(09)00128-0 [pii] 10.1016/j.jaut.2009.10.001 (2009).

6 Xu, Y. *et al.* Pleiotropic IFN-dependent and -independent effects of IRF5 on the pathogenesis of experimental lupus. *Journal of immunology* **188**, 4113-4121, doi:10.4049/jimmunol.1103113 (2012).

7 Yang, L., Feng, D., Bi, X., Stone, R. C. & Barnes, B. J. Monocytes from Irf5-/- mice have an intrinsic defect in their response to pristane-induced lupus. *Journal of immunology* **189**, 3741-3750, doi:10.4049/jimmunol.1201162 (2012).

8 Partida-Sánchez, S. *et al.* Chemotaxis and Calcium Responses of Phagocytes to Formyl Peptide Receptor Ligands Is Differentially Regulated by Cyclic ADP Ribose. *The Journal of Immunology* **172**, 1896-1906, doi:10.4049/jimmunol.172.3.1896 (2004).

9 Lischke, T. *et al.* CD38 controls the innate immune response against Listeria monocytogenes. *Infection and immunity* **81**, 4091-4099, doi:10.1128/IAI.00340-13 (2013).

10 Bannenberg, G. L. *et al.* Molecular circuits of resolution: formation and actions of resolvins and protectins. *Journal of immunology* **174**, 4345-4355 (2005).

11 Lee, P. Y. *et al.* Type I interferon modulates monocyte recruitment and maturation in chronic inflammation. *Am J Pathol* **175**, 2023-2033, doi:10.2353/ajpath.2009.090328 (2009).

12 Barr, T. A., Brown, S., Ryan, G., Zhao, J. & Gray, D. TLR-mediated stimulation of APC: Distinct cytokine responses of B cells and dendritic cells. *Eur J Immunol* **37**, 3040-3053, doi:10.1002/eji.200636483 (2007).

13 Untergasser, A. *et al.* Primer3--new capabilities and interfaces. *Nucleic Acids Res* **40**, e115, doi:10.1093/nar/gks596 (2012).
